# Altered pathways and targeted therapy in double hit lymphoma

**DOI:** 10.1186/s13045-022-01249-9

**Published:** 2022-03-18

**Authors:** Yuxin Zhuang, Jinxin Che, Meijuan Wu, Yu Guo, Yongjin Xu, Xiaowu Dong, Haiyan Yang

**Affiliations:** 1 Department of Lymphoma, The Cancer Hospital of the University of Chinese Academy of Sciences (Zhejiang Cancer Hospital), Institute of Basic Medicine and Cancer (IBMC), Chinese Academy of Sciences, Hangzhou, People’s Republic of China; 2 Hangzhou Institute of Innovative Medicine, Institute of Drug Discovery and Design, College of Pharmaceutical Sciences, Zhejiang University, Hangzhou, People’s Republic of China; 3 Innovation Institute for Artificial Intelligence in Medicine of Zhejiang University, Hangzhou, People’s Republic of China; 4 Department of Pathology, The Cancer Hospital of the University of Chinese Academy of Sciences (Zhejiang Cancer Hospital), Institute of Basic Medicine and Cancer (IBMC), Chinese Academy of Sciences, Hangzhou, People’s Republic of China; 5 Cancer Center, Zhejiang University, Hangzhou, People’s Republic of China

**Keywords:** Double hit lymphoma, Diffuse large B-cell lymphoma, Genetic alterations, Targeted therapy, Chemotherapy

## Abstract

High-grade B-cell lymphoma with translocations involving *MYC* and *BCL2* or *BCL6*, usually referred to as double hit lymphoma (DHL), is an aggressive hematological malignance with distinct genetic features and poor clinical prognosis. Current standard chemoimmunotherapy fails to confer satisfying outcomes and few targeted therapeutics are available for the treatment against DHL. Recently, the delineating of the genetic landscape in tumors has provided insight into both biology and targeted therapies. Therefore, it is essential to understand the altered signaling pathways of DHL to develop treatment strategies with better clinical benefits. Herein, we summarized the genetic alterations in the two DHL subtypes (DHL-BCL2 and DHL-BCL6). We further elucidate their implications on cellular processes, including anti-apoptosis, epigenetic regulations, B-cell receptor signaling, and immune escape. Ongoing and potential therapeutic strategies and targeted drugs steered by these alterations were reviewed accordingly. Based on these findings, we also discuss the therapeutic vulnerabilities that coincide with these genetic changes. We believe that the understanding of the DHL studies will provide insight into this disease and capacitate the finding of more effective treatment strategies.

## Background

Diffuse large B cell lymphomas (DLBCLs) are aggressive, heterogeneous neoplasms with distinct biological, pathological and clinical characteristics, representing ~ 25% of non-Hodgkin lymphomas (NHL) [1]. DLBCLs harboring translocation of *MYC*(8q24) and *BCL2*(18q21) or/and *BCL6*(3q27) displays a highly aggressive profile, including high incidence of advanced disease at diagnosis, and poor response to up-front R-CHOP therapy (5-year overall survival [OS] and progression-free survival [PFS] rates of 27% and 18%, versus 71% and 65% in other DLBCLs) [2]. Owing to their unique genomic features, biological behaviors, and prognosis, the subtypes were classified as a new category in the 2016 revision of the World Health Organization (WHO) classification for lymphoma. They were termed high-grade B-cell lymphoma (HGBL) with translocations involving *MYC* and *BCL2* and/or *BCL6*, also called double hit lymphoma (DHL) or triple hit lymphoma (THL) [3]. DHL involves the gene translocation of MYC and BCL2 (DHL-BCL2) or MYC and BCL6 (DHL-BCL6), while THL involves the translocation of all these three genes.

Given inferior outcomes with current therapies in DHL patients, further understanding disease pathology is of great importance to developing better regimens. Herein, we reviewed current clinical management, genetic alterations, and their physiological functions, as well as novel agents in development, hoping to enlighten future exploration in DHL. Since the majority of histological subgroups of DHLs are DLBCLs [3], this review focuses on DHLs fulfilling DLBCL histology. These DHLs were termed as DHL-BCL2 (*MYC* and *BCL2* translocation) and DHL-BCL6 (*MYC* and *BCL6* translocation) in this review.

## Diagnosis, prognosis, and current treatment

### Diagnosis of DHLs

DHLs are identified in 5–15% DLBCLs [3]. Diagnosis of DHLs requires identification of translocations of *MYC*(8q24) and *BCL2*(18q21) or/and *BCL6*(3q27), which is usually accomplished by fluorescent in-situ hybridization (FISH) [4]. Up to now, no consensus has been established as to which patients should have FISH tests. Some institutions recommend FISH to all DLBCL patients (routine testing), whereas others only recommend it to certain patients (selective testing) due to high cost and low prevalence [5]. In the centers performing selective testing, decisions are made based on patients’ pathologic characteristics, including MYC and **BCL2** protein expression, Ki-67 percentage and cell-of-origin (COO) subtype, etc. [6, 7].

Based on COO, DLBCL could be classified into GCB and non-GCB via immunohistochemistry test (IHC), which is the most common technique with low cost used in clinic. In academic institutions, gene expression profiling (GEP) could also be used to further classify DLBCL into GCB, ABC and unclassified subtypes. Due to different prevalence of DHL-BCL2 and DHL-BCL6 in these COO subtypes, COO diagnosis could help the decision of selective testing. However, the consistency between IHC and GEP is not 100%. Sriram Balasubramanian and colleagues reviewd 910 patients’ IHC and GEP results from PHOENIX study. IHC result was 82.7% concordant with that of GEP. Due to the cost issue, IHC is currently more accessible.

### Prognosis and risk stratification

Even within the same molecular group, the clinical outcome can be very different. Though conflicting results were yielded from different studies when associating *MYC* partner to prognosis [8–11], IG translocation partner of MYC likely indicates inferior outcome, especially in DHLs [12]. Additionally, DLBCL co-expressing MYC and **BCL2** (double expressor lymphomas (DEL)) are associated with significantly shorter OS and PFS in both DHL and non-DHL [2, 12–14]. Interestingly, DHL cases without concurrently expressing MYC and **BCL2** had more favorable outcomes than DEL after ASCT [13]. Sha et al. defined a molecular high-grade (MHG) group in DLBCL patients based on gene expression profile [15]. The 3-year PFS of the MHG group and others treated with bortezomib plus R-CHOP were 37% and 72%, respectively. Intriguingly, only half of the MHG group was diagnosed with DHL, whereas the other half demonstrated inferior outcomes compared to the non-MHG group, irrespective of their translocation status. The majority of DHLs were classified as MHG. However, no signs of worse outcomes were observed in DHLs lacking MHG features.

When compared to DHL-BCL2/THL, DHL-BCL6, the minority of all DHL, possess distinct features in many aspects. In contrast to DHL-BCL2/THL which are distinct GCB subtypes, DHL-BCL6 can be found in lymphoma with various COO including GCB, ABC, and unclassified. DHL-BCL6 has a lower proportion of MYC + by IHC compared to DHL-BCL2/THL, whereas DHL-BCL2/THL share similar morphology (high-grade with a starry sky appearance) and immunophenotype (CD10+, MCY + BCL2+, and MUM1) [16, 17]. Also, DHL-BCL6 tends to have a less cytogenetic complexity [18]. Recent studies indicate that compared to MYC/BCL2 lymphoma, lymphoma with BCL6 translocation shas a distinct molecular profile [19–23], suggesting a distinct molecular group from the DHL-BCL2/THL, the majority of DHL. Therefore, more study should be done to investigate the molecular mechanism and category of these two subtypes.

In terms of prognosis, DHL-BCL2 and THL have similar OS [17, 24]. As for DHL-BCL6, some studies suggest that this subtype is more aggressive, frequently involving extranodal sites, and has worse OS compared to DHL-BCL2 [17, 18, 24]. However, there are contradicting results indicating that DHL-BCL6 has a similar, or even better prognosis than DHL-BCL2 [24, 25]. The conflicting conclusion may be the result of a limited sample population (~ 10 in most studies).

The prognosis is different within the DHL-BCL2 group. Parallelly, a 104-gene double-hit signature (DHITsig) developed by Ennishi et al. distinguished a cohort of GCB-DLBCLs (DHITsig positive) from other GCB-DLBCLs (DHITsig negative) [26]. DHITsig positive group was composed of 48% non-DHL/THL-BCL2s and 52% DHL/THL-BCL2s (88% of all DHL/THL-BCL2s). DHITsig positive patients had dismal outcomes after being treated with R-CHOP in terms of DSS (BioElectric Massage) (pos vs neg, 63% vs 90%), 5-year PFS (pos vs neg, 53% vs 79%), and OS (pos vs neg, 60% vs 83%). Several literature highlighted the importance of risk stratification depending on molecular features like GEP (gene expression profile), instead of merely genetic translocation status, to avoid missing out on a cluster of high-risk patients while exposing low-risk ones to excessive therapies [27, 28].

### Current chemotherapy

The standard regimen for DHL has not been established yet. When clinical trials are not available, chemotherapy is the first choice. Regimens, like R-CHOP, R-DA-EPOCH, R-Hyper-CVAD, and R-CODOX-M/IVAC, have been clinically evaluated in Table 1 [29].

**Table 1:** Comparison of treatment regimens and outcome of DHL in retrospective studies

Treatment	Response	PFS/PFS/EFS (months)	OS (months)	References
R-CHOP (*n* = 35) vs intense regimens (DA-EPOCH-R (*n* = 81), R-hyperCVAD (*n* = 32), R-CODOX-M/IVAC (*n* = 11))	Patients with DHL who achieved first complete remission	3-year RFS rate: R-CHOP 56% vs Intense Regimens 88% 3-year RFS rate: DA-EPOCH-R 88%, R-hyperCVAD 87%, and R-CODOX-M/IVAC 91%	3-year OS rate: R-CHOP 77% vs Intense Regimens 90% (*P* = 0.13) 3-year OS rates: DA-EPOCH-R 87% vs R-hyperCVAD 90% vs R-CODOX-M/IVAC 100%, *P* = 0.57	Landsburg et al. [36]
R-CHOP (*n* = 5) vs R-CODOX-M/IVAC (*n* = 25)	CR rate: R-CHOP 0% vs R-CODOX-M/IVAC 36% ORR: R-CODOX-M/IVAC 80%	2-year PFS rate: R-CODOX-M/IVAC 47%	2-year OS rate: R-CODOX-M/IVAC 61%	Sun et al. [37]
R-CHOP (*n* = 100) vs R-DA-EPOCH (*n* = 28) vs R-Hyper CVAD (*n* = 34)	CR rate: R-CHOP 40% vs R-DA-EPOCH 68% (*P* = 0.017 vs. R-CHOP) vs R-Hyper CVAD 68% (*P* = 0.011 vs. RCHOP)	2-year EFS rate: R-CHOP 25% vs R-DA-EPOCH 67% vs R-Hyper CVAD 32% 3-year EFS rate: R-CHOP 20% vs R-DA-EPOCH 67% (*P* = 0.004 vs. R-CHOP) vs R-Hyper CVAD 32% (*P* > 0.05 vs. R-CHOP)	2-year OS rate: R-CHOP 41% vs R-DA-EPOCH 76% vs R-Hyper CVAD 44% 3-year OS rate: R-CHOP 35% vs R-DA-EPOCH 76% (*P* = 0.057 vs. R-CHOP) vs R-Hyper CVAD 40% (*P* > 0.05 vs. R-CHOP)	Oki et al. [33]
R-CHOP (*n* = 100) vs intense regimens (R-DA-EPOCH (*n* = 65), R-Hyper CVAD (*n* = 64), R-CODOX-M/IVAC (*n* = 42))	CR rate: DA-EPOCH-R was significantly higher compared with R-CHOP, R-CODOX-M/IVAC, or “other/multiple” regimens	Median PFS: R-CHOP 7.8 months vs intense regimens 21.6 months	OS in intensive chemotherapy (R-DA-EPOCH, R-Hyper CVAD, R-CODOX-M/IVAC) is higher than R-CHOP	Petrich et al. [32]
R-CHOP (*n* = 19) vs R-Hyper CVAD (*n* = 28)	NA	PFS: R-CHOP vs R-Hyper CVAD, *P* > 0.05	OS: R-CHOP vs R-Hyper CVAD, *P* > 0.05	Li et al. [38]

When treated with R-CHOP, DHLs were found to be associated with inferior OS compared to other DLBCLs (median OS: 13 months vs 95 months; 3-years OS: 46% vs 75%, HR 3.04, *P* = 0.002) [30]. Besides, when retrospectively evaluating application of IFRT(involved-field radiation therapy), R-CHOP and more intensive immunochemotherapy in patients with limited-stage (LS) DHL, similar PFS and OS were observed throughout the treatment groups (IFRT vs no IFRT, and R-CHOP vs more intensive immunochemotherapy) [31].

Several retrospective studies and meta-analyses have compared R-CHOP to more intensive regimens in DHL patients. Petrich et al. analyzed 311 newly diagnosed DHL patients, receiving R-CHOP (31%), R-DA-EPOCH (21%), R-HyperCVAD (21%), and R-CODOX-M/IVAC (14%). They found that intensive regimens were associated with significantly improved PFS, but not OS [32]. Oki et al. found that R-EPOCH was associated with longer EFS (HR = 0.37, *P* = 0.008) compared to R-CHOP, while R-HyperCVAD did not bring more benefit [33]. A meta-analysis compared the survival outcomes in DHL patients receiving more intensive regimens (R-DA-EPOCH, *n* = 91, and R-Hyper CVAD or R-CODOX-M/IVAC, *n* = 123) versus R-CHOP (*n* = 180) [34]. First-line treatment with R-EPOCH significantly reduced progression risk compared to R-CHOP (relative risk reduction of 34%; *P* = 0.032), but not OS. In all, these studies supported use of more intensive regimens like R-DA-EPOCH over R-CHOP when treating DHL patients. However, a retrospective multicenter study conducted on 90 cases of DEL patients showed no survival benefit from DA‐EPOCH‐R comparing to R-CHOP [35]. These contradicted results indicate that prospective studies are in need.

### Transplantation

Allogeneic/autologous stem cell transplant (allo-SCT/auto-SCT) is a potentially curative option for many hematological malignancies and has been evaluated in the DHL cohort. However, most studies suggest that SCT may have a dismal effect on DHL patients.

In a multicenter study involving 311 DHL patients in North America, the response of patients receiving SCT (allo-SCT or auto-SCT) after induction chemotherapy, which includes R-CHOP, R-HyperCVAD/MA, DA-EPOCH-R, or R-CODOX-M/IVAC, as well as other non-rituximab regimens (5%) were documented. After the first CR, the median OS is between the SCT group(*n* = 39) and observation group(*n* = 112) did not have a significant difference [32]. Similar results were obtained by independent studies in 2014(*n* = 129),2017(two studies, *n*1 = 159, *n*2 = 117) and 2021(*n* = 160). Auto-SCT after a front line or intensive chemotherapy in DHL patients did not affect 3-year PFS or OS after first CR [13, 33, 36, 39]. On the other hand, these studies again emphasize the importance of intensive therapy compared to front-line therapy in improving the 3-year RFS [36]. Nevertheless, according to a 2016 trial, patients with DEL or MYC overexpression may benefit from auto-SCT following CHOP/− + R treatment, presenting a lightly longer PFS and OS [40].

## Genetic alterations in DHL

DHL is accompanied by genetic and molecular features that distinguish them from other DLBCL and Burkitt. With several recent studies focusing on the genetic classification of DLBCL [19–22], we summarized genetic alterations that intensively or exclusively coincides with the translocation of *MYC* and ***BCL2*** and/or ***BCL6***, and characterized consequent functional disturbance.

### Genetic alterations in DHL-BCL2

From previouspublications, DLBCL subgroups C3 [20], EZB(-DHIT+) [21, 22], **BCL2** [19], and MYC/BCL2-DH [23] shared similar genetic profiles and unanimously encompassed dual translocations of *MYC* and *BCL2*.

**Fig. 1:**
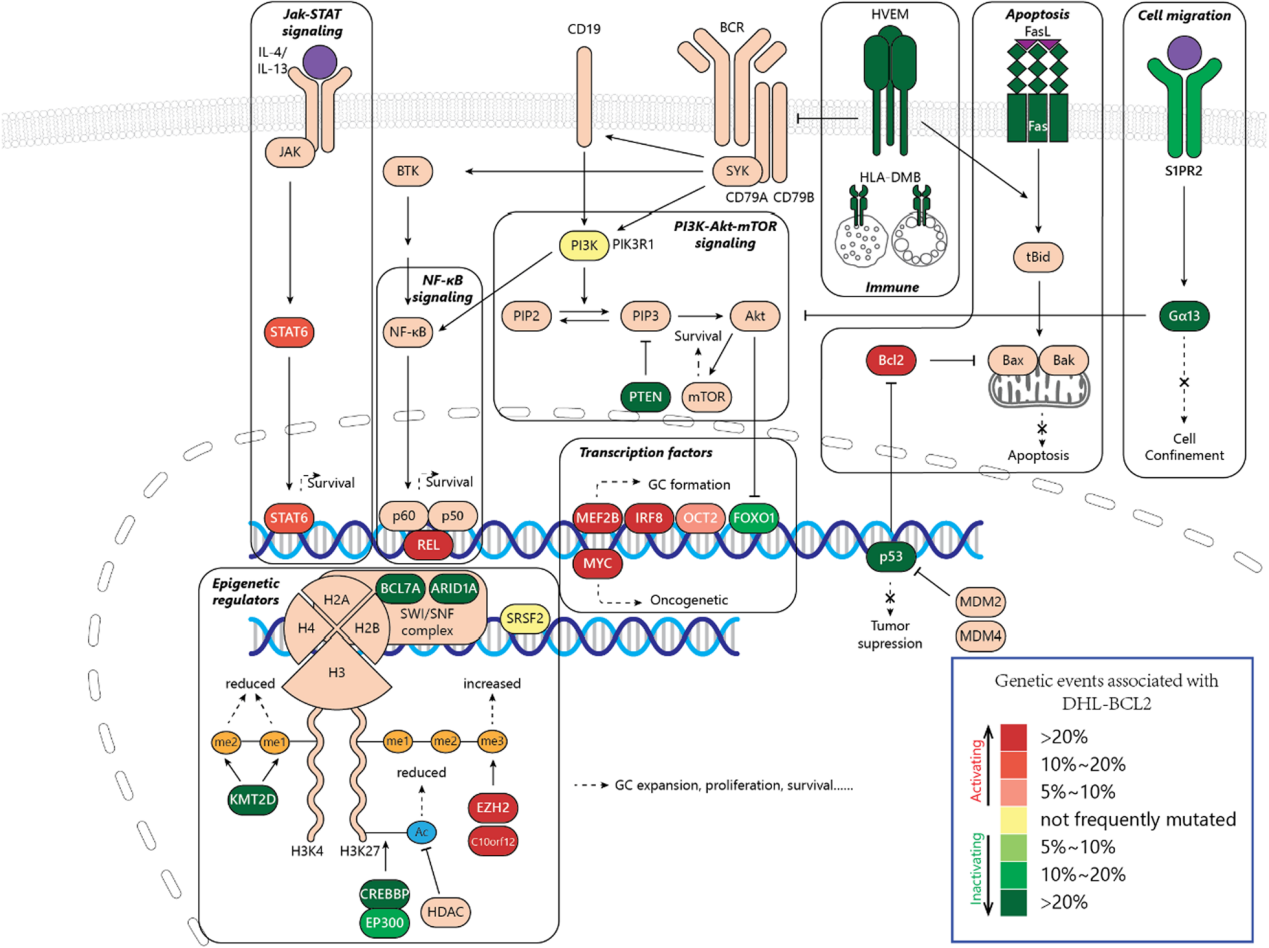
Genetic and pathways alterations involved in DHL-BCL2

Herein, we summarized genetic similarities of these subgroups and combined them into one group called DHL-BCL2 (Fig. 1). DHL-BCL2 lymphomas were exclusively germinal center lymphomas with aberrant GC formation. DHL-BCL2 lymphomas were exclusively germinal center lymphomas [16, 41], with frequent genetic alterations associated with the formation of GC [42–45]. The primary genetic lesions are alterations associating epigenetic regulators like *KMT2D*, *EZH2,* and *CREBBP*/*EP300*. DHL-BCL2 also exhibited an anti-apoptotic and pro-survival profile, with aberrant activation of BCL2 and PI3K-mTOR signaling. Inactivating lesions of the S1PR2-Gα13 axis contributes to tumor cells dissemination, while damaged TNFRSF14 leads to increased CD40 signaling. Other proto-onco mutations also occur in this subtype, including activation of MYC, JAK-STAT, and NF-κB signaling, as well as suppressing p53 pathway. Gene alteration frequency and categorizing foundation were summarized in Table 2.

**Table 2:** Genetic aberrations associated with DHL-BCL2 [19–23]

Category	Gene (frequency): cluster
Transcription factors	***IRF8*** (22%): E+, C, B, D(T); ***MEF2B*** (21%): E+, C, B; ***MYC*** (32%): E+, C3; ***FOXO1*** (13%): E+, B; ***POU2F2*** (9%): C, B; ***EBF1*** (27%): E+
Epigenetic regulators	***KMT2D*** (59%): E+, C, B, D(T); ***EZH2*** (55%): E+, C, B, D; ***CREBBP*** (53%): E+, C, B, D(T); ***EP300*** (11%): E+, B; ***BCL7A*** (28%): E+, B; ***ARID1A*** (27%): E+, T; ***C10orf12*** (27%): E+; ***HIST1H1D*** (16%): B; ***SRSF2*** (4%): B;
Apoptosis	***BCL2*** (70%): E+, C, B, D(T); ***FAS*** (38%): E+
PI3K-mTOR	***PTEN*** (22%): E+, C; ***PIK3R1*** (4%): B
NF-κB	***REL*** (40%): E+;
JAK-STAT	***STAT6*** (19%): E+, C, B;
P53	***TP53*** (44%): E+;
Immune	***TNFRSF14*** (50%): E+, C, B, D(T); ***HLA-DMB*** (22%): E+
Cell migration	***GNA13*** (26.30%): E+, C, B, D(T); ***SIPR2*** (11%): E+
Others	***Chrom.12p*** (28%): E+, C; ***GNAI2*** (13%): E+, C; ***DDX3X*** (33%): E+; ***10q23.31*** (24%): C; ***Chrom.21*** (20%): E+; ***HVCN1*** (13%): C

*E* + : EZB-DHIT + cohort, *C* C3 cohort, *B* **BCL2** cohort, *D(T)* MYC/BCL2-DH(TH) cohort

#### Alterations relating cellular differentiation and transcription factors

In DHL-BCL2, disrupted the transcriptional process of B cell development may contribute to dysregulated GC development, altered apoptosis profile, and eventually lymphomagenesis.

Both interferon regulatory factor8 (IRF8) and myocyte enhancer factor 2B (MEF2B) are vital transcription factors in GC formation and frequently mutated in DHL-BCL2. In normal B cells, IRF8 transactivates *BCL6* and *AICDA* [46] and prevents B cells from p53-induced apoptosis by up-regulating MDM2 [47]. MEF2B modulates the expression of a series of genes from the cell cycle (*CCND3*), differentiation (*BCL6*) to apoptosis (*TP53* and *BCL2*) [48]. They are preferentially expressed and frequently altered in B cell lymphomas of GC origin like DLBCL and follicular lymphoma [20–22, 49–53]. The mutation of *MEF2B*^*D83V*^ contributes to lymphomagenesis by enlarging GC formation, dysregulating level of *BCL6* [50] and other gene targets [48], while *IRFB* mutation and translocation were also believed to promote lymphomagenesis [46, 51].

MYC protein plays a crucial role in cellular processes, including growth, proliferation, metabolism, and biosynthesis [54, 55]. Besides *MYC* translocation, mutation and amplification are also repeatedly found in DHL-BCL2 [22, 56], leading to dysregulated MYC activity and oncogenic signals. *MYC* translocation adjacent to IG enhancer [8] and *MYC* amplification engender elevated MYC expression [57, 58]. *MYC* mutation, on the other hand, has divergent impacts on MYC expression and clinical outcomes [56]. However, the implications of most mutations have not yet been elucidated.

Forkhead box O1 (*FOXO1*) fosters B cell differentiation [59], and is expressed and recurrently mutated in DLBCLs [19, 21, 22, 60, 61]. FOXO1 is restricted by PI3K signaling [62], Its absence abrogates toxicity induced by SYK and AKT inhibitor [63]. It has been reported that *FOXO1* mutation is associated with inferior prognosis [60], refractory and relapsed GCB-DLBCL [64], as well as correlations with *TP53* and *BCL2* mutation [64, 65]. Other transcription factors, like octamer-binding protein 2 (OCT2) which is encoded by POU Class 2 Homeobox 2(*POU2F2*), has been proposed as a therapeutic target due to its role in promoting B cell proliferation in GC [66], inducing the IL-6 expression [67] and regulating SYK expression [68].

#### Alterations relating apoptosis

The BCL2 family is constituted of three subfamilies: anti-apoptotic proteins (including **BCL2**, BCL-XL, BCL-W, MCL-1, BFL-1/A1, and BCL-B/BCL2L10), pro-apoptotic BH3-only proteins, and pro-apoptotic effector proteins (BAX and BAK). BH3-only proteins respond to cytotoxic stimuli or extrinsic pathway signals (FAS and TNFR1), expose their BH3 domain and subsequently neutralize anti-apoptotic proteins or directly activate BAX and BAK, to initiate oligomerization, and eventually to trigger caspases-dependent apoptosis [69].

Besides *BCL2* translocation juxtaposed to IGH enhancer, *BCL2* mutation is also enriched in this subtype [19–23, 70]. Non-synonymous mutations of *BCL2* were primarily distributed in three domains: the p53 binding site of flexible loop domain (FLD), the BH4 domain, and the region between BH3 and BH1 [70, 71], thereby promoting its anti-apoptotic effect by disrupting its interaction with p53 [72], inositol 1,4,5-trisphosphate receptor (IP(3)R) [73] and BAX [74], respectively. Moreover, recurrent mutations in the BH3 domain of *BCL2* contribute to the resistance of venetoclax [75].

After stimulation by FASL, FAS(CD95) can induce apoptosis and contribute to immune contraction and cell migration [76]. In EZB-DHITsig+, mutation and deletions are frequently observed in the intracellular death domain of *FAS*, resulting in apoptotic function loss and further inferior survival [77, 78].Additionally, genetic signatures, which were associated with the depletion of immune and stromal cells and the augment of tumor cells, were enriched in the EZB-DHITsig+ cluster with *FAS* alteration [78].

#### Alterations relating to epigenetic regulators

Alterations of epigenetic regulating genes are the most prominent in DHL-BCL2 [19–21, 23, 79]. Some have been considered promising therapeutical targets.

Histone methylation and demethylation occur at the lysine residue of histone H3 or H4 to different degrees (mono-methylation(me1), di-methylation (me2), or trimethylation (me3)), depending on status of active or inactive chromatin. Upon histone methylation, proteins with methyl-binding domains recognize the methylated lysine and initiate transcriptional activation or repression [80, 81].

KMT2D is a histone lysine methyltransferase that catalyzes the mono- and di-methylation of lysine 4 on histone H3(H3K4). Premature stop codons upstream of the enzymatic region can be introduced by deletion and nonsense mutation, leading to KMT2D inactivation. Missense mutations at C-terminal domains and other possible mechanisms contribute to impaired methyltransferase activity without *KMT2D* alteration [19–23, 43]. KMT2D lesions cause reduced H3K4 methylation and altered transcriptome, which mainly affects genes regarding cell cycle regulation, anti-apoptosis, and pro-apoptosis. Such process also contributes to increased GC formation, pro-proliferative cellular profile, inhibition of tumor-suppressive pathways, accelerated lymphoma development, and even malignant transformation when cooperate with BCL2 [43, 82].

As for H3K27, hypermethylation may cause lymphomagenesis. EZH2 is a component of polycomb repressive complex 2 (PRC2) which methylates H3K27 and transcriptionally represses genes relating to the cell cycle checkpoint [83]. Missense mutations of EZH2 are enriched at Y641 in the SET domain and have been identified in different patient cohorts and cell lines [19–23, 83–85]. Although EZH2 with mutant Y641 showed a reduced ability to directly tri-methylate H3K27 [85], global cellular H3K27me3 level containing EZH2 Y641 is still remarkably promoted owing to the increased substrate preference of EZH2 for H3K27me2 and increased EZH2 stability [84, 86]. *EZH2* mutation and aberrantly hypermethylated H3K27 suppress differentiation and promote proliferation of GC B cells. It also alters the expression of *EZH2*, *MYC,* and genes relating to B cell regulation decreases MHC expression, and reduces T cell infiltrate in the tumor microenvironment. When cooperating with BCL2, MYC, or BCL6/BCOR complexes, *EZH2* mutation promotes lymphomagenesis [87–91]. C10orf12 was also identified to interact with histone methyltransferase PRC2. The interaction enhances activity and substrate preference of PRC2, and thereby upregulate cellular H3K27me3 levels [92].

Normally, histone acetylation dissociates its interaction with DNA, leading to genetic transcription. CREBBP and EP300 are transcriptional coactivators comprising a catalytic histone acetyl-transferase domain [93]. *CREBBP* and *EP300* have high sequence homology, yet their function in GC development turns out to be different. Deletion of both genes results in abrogation of GC formation [94]. In DHL-BCL2, *CREBBP* alteration (including deletions and mutations) is more frequent than that of *EP300* [19–23, 95]. Loss-of-function of *CREBBP* leads to loss of H3K27ac at putative enhancers, expanded GC compartment, interfered plasma cell differentiation, indirective induction of MYC level, inactive p53 acetylation, proliferative profile, and MHC II deficiency of B cells. It also induces lymphomagenesis in an HDAC3 dependent manner, especially when cooperates with dysregulated BCL2 [42, 96–98]. Mutation of *EP300* hardly overlaps with that of *CREBBP* [99]. EP300 function repression due to mutation or BCL6 suppression reduces H3K27ac, induces cell growth, and impairs the effect of BCL6 inhibitors and anti-CD20 antibodies to DLBCL cells [99–101]. Additionally, *CREBBP* and *EP300* mutations contribute to the polarization of tumor-associated macrophages by increasing expression of CCL2, CSF1, and IL-10 via the Notch pathway [99].

SWI/SNF chromatin remodeling complexes regulate the transcriptional accessibility of DNA by mobilizing histone octamers to adjacent DNA positions [102, 103]. Point mutations of *BCL7A*, a subunit of SWI/SNF complexes, is enriched in the first splice donor and results in BCL7A inactivation, damaging BCL7A binding to SWI/SNF complex [19, 21, 22, 104], while wild *BCL7A* exhibits a tumor-suppressing role [104]. *ARID1A* is another subunit of SWI/SNF chromatin remodeling complexes. Deletions of *ARID1A* may affect the regulatory effect on CDKN1A, and promote proliferation and the ubiquitin–proteasome-dependent degradation of ARID1A [105]. The mutation of *ARID1A* and *FOXO1* is also associated with loss of HLA-C [106]. It is worth noting that the *ARID1A*-deficient cancers are susceptible to the perturbance of homolog *ARID1B*, indicating that synthetic lethality may be a possible therapeutic option in these specific tumors [107].

Additionally, the P95 mutant of splicing factor SRSF2 identified in DHL-BCL2 alters the binding and global splicing event of RNA, thus affecting the regulation of gene expression and leading to abnormal hematopoiesis [108, 109].

#### Alterations relating to oncogenic pathways

1. Alterations relating PI3K-Akt-mTOR and BCR-NF-κB pathway

In GCB-DLBCLs, tonic BCR signaling leads to proliferation via the activation of NF-κB [110]. c-Rel, a protein of the NF-κB family, forms homo- or heterodimer with other NF-κB subunits after upstream stimuli, and translocate to the nucleus to transcriptionally activate genes associated with survival and proliferation [111]. c-Rel is encoded by *REL* whose amplification has early been identified as a genetic alteration associated with GCB-DLBCL [112] and is recently recognized as an EZB-related event [21, 22]. However, although this amplification arouses increased abundancy of c-Rel mRNA [113], nuclear c-Rel level and NF-κB activity do not correlate with *REL* amplification [113–115].

The activation of co-receptor of BCR, CD19, and SYK kinase can also trigger the signaling of the PI3K-Akt-mTOR pathway, which promotes cell survival [116]. As a negative regulator of Akt, PTEN loss caused by deletion and truncating mutation promotes tonic BCR signaling, Akt activation, and cell proliferation [20, 22, 110, 117].

(2) Alterations relating JAK-STAT pathway

The JAK-STAT signaling cascade consists of three main components: a cell surface receptor, a JAK, and two STAT proteins. Once the cytokine binds to its receptor, the JAK family will be phosphorylated, resulting in the recruitment of phosphorylated and dimerized STAT proteins [118]. Subsequently, STATs are translocated to the nucleus and transcriptionally participate in cell proliferation, survival, angiogenesis, and immunity [119].

Amplification of *STAT6* in DHL-BCL2 enhances JAK-STAT signaling [20, 22]. Missense mutations result in repetitive alterations in the DNA-binding domain of STAT6, especially at D419, which is both prevalent in DHL-BCL2 and R/R DLBCL [19, 64]. STAT6-D419 mutations were recognized as the gain of function mutations owing to the increased nuclear localization of STAT6, upregulated STAT6-targeted gene expression, and increased sensitivity to JAK2 inhibitors of STAT6-D419-mutated lymphomas [120–122].

(3) Alterations relating p53

P53 is a tumor-suppressing protein encoded by *TP53*, which transactivates genes regarding apoptosis and cell cycle. It can be degraded by MDM2-mediated ubiquitination and proteasomal degradation and repressed by sumoylation and neddylation [123]. Mutation of *TP53* occurs in ~ 20% of all DLBCLs [21, 124], preferentially in DHLs with *MYC* and *BCL2* rearrangements [22, 125]. In DHL-BCL2, most mutations are located in the DNA-binding core domain of *TP53*, posing a deleterious effect on p53 function [125] since the transcription activity of p53 is requisite for its tumor-suppressing ability [126]. In DHLs, DHITsig positive DLBCLs [127] and the rest of DLBCLs, as well as other lymphoid malignancies mutated *TP53* status [123, 124] are correlated with inferior prognosis.

(4) Other alterations relating to oncogenic pathways

Though they have not been fully elucidated, some of the mutations of *GNAI2* are orthologous to gain-of-function mutations of *GNAI3*, indicating an oncogenic effect [128]. Also, activating mutations of *GNAI2* at the GTP-binding domain may result in aberrant MAPK/ERK signaling [129]. *DDX3X* is a tumor-suppressing gene that is recurrently mutated in Burkitt lymphoma and EZH-DHIT + B cell lymphomas, and such mutation results in the truncation or loss of DDX3X [22, 130]. In NK/T cell lymphomas, DDX3X mutant activates NK-κB and MAPK signaling, while losing the effect in inducing apoptosis, inhibiting the activation of ERK and RelB compared to its WT counterpart [131]. HVCN1 is also frequently mutated and truncated, which might induce stronger BCR-dependent signaling, proliferation, and migration [132].

#### Alterations relating to immune escape

Recurrent *TNFRSF14* mutations in DHLs lead to reduced expression of HVEM [19, 20, 22, 23, 78], a TNFR family protein and tumor suppressor which inhibits BCR signaling, abnormal stroma activation, and CD40 signaling through BTLA stimulation [133, 134]. Also, when triggered by LIGHT, it will render tumor B cells higher immunogenicity and sensitivity to FAS-induced apoptosis [135].

#### Alterations relating to cell migration

S1PR2-Gα13 (encoded by *SIPR2* and *GNA13*, respectively) signaling regulates cell growth, confinement, and localization of B cells to GC through inhibiting Akt phosphorylation under normal physiological conditions [136], while its dysregulation promotes lymphomagenesis [136–139]. *GNA13* mutation, which often co-occurs with *BCL2* rearrangement [19, 20, 22, 23, 70, 79], contributes to disrupted GC architecture in lymph nodes and disseminates GC B cells to systematic circulation [137]. Furthermore, loss of function mutation of *GNA13* impairs its ability in suppressing BCL2 expression [140].

### Genetic alterations in DHL-BCL6

Compared to their counterparts, DHLs with BCL6 translocations are relatively minor described. As reported in multiple centers, DHL-BCL2 are mainly GCB subtypes [19, 20, 22], while GCB, ABC, and undefined lymphoma can all be identified in DHL-BCL6 [141]. Upon previous publications, genetically categorized subtypes C1 [20], BN2 [21], NOTCH-2 [19], and BCL6-DH [23] are all accompanied by the translocation of BCL6, suggesting possible similarities between DHL-BCL6 and these published subtypes.

**Fig. 2:**
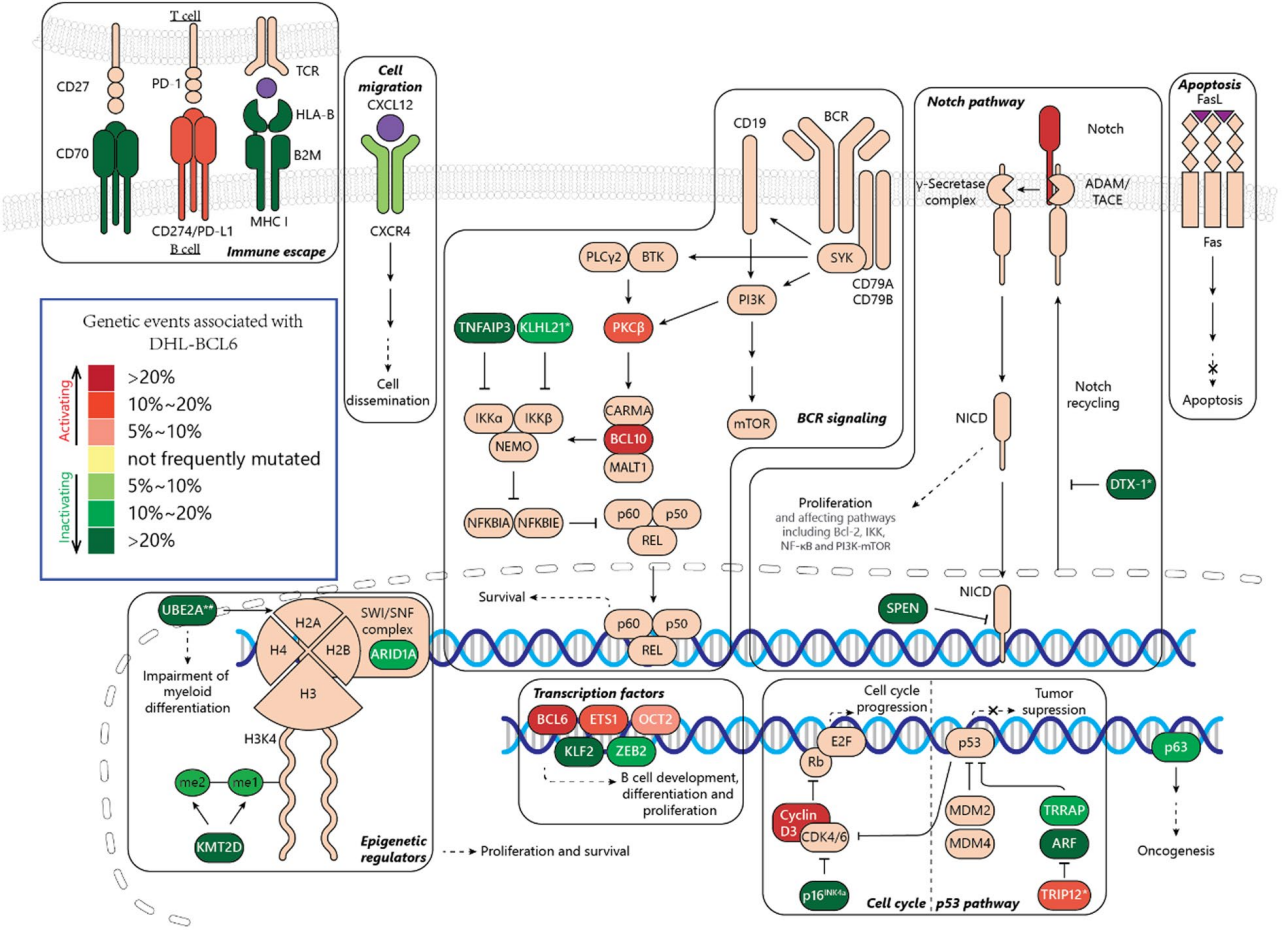
Genetic and pathways alterations involved in DHL-BCL6. Note: *genes associated with the ubiquitin–proteasome system; #: the consequence of *UBE2A* mutation is not fully illustrated, might be functionally damaging [142]

In this review, we summarized these genetic subtypes as DHL-BCL6. Genetic alterations in DHL-BCL6 closely resemble genetic events in marginal zone lymphomas (Fig. 2) [19–22, 143]. Genetic lesions in this subtype are associated with the disruption of immune escape, Notch signaling, and ubiquitin–proteasome degradation system. Pro-proliferative signatures, including cell cycle genes mutations, BCR and NF-κB signaling activation, p53 pathways suppression, are also observed in this subtype. Detailed annotations were presented in Table 3.

**Table 3:** Genetic aberrations associated with DHL-BCL6 [19–23]

Category	Gene (frequency): cluster
B cell development and differentiation	***BCL6*** (69%): B, C, N; UBE2A*(24%): B, C; ***KLF2***(22%): B; ***ETS1***(17%): B; *ZEB2*(14%): C; ***POU2F2***(6%): N
BCR and NF-κB signaling	***BCL10***(28%): B, N, C, D; ***TNFAIP3***(29%): B, C, N; ***PRKCB*** (16%): B; KLHL21*(12%): B
Notch	***NOTCH2***(31.3%): B, N, C, D; ***SPEN*** (21%): B, C, N; DTX1*(38%): B, C
Cell cycle	***CCND3***(22%): B, N; ***CDKN2A*** (29%): N
P53	***TP63***(15%): B, C; TRIP12*(12%): B; ***TRRAP*** (10%): B
Epigenetic regulator	***HIST1H2BK*** (16%): B, C; ***KMT2D*** (41%): N; ***HIST1H1D*** (16%): B; ***ARID1A*** (14%): N
Immune escape	***CD70***(25%): B, C, N; ***B2M*** (28%): C, N; ***HLA-B*** (23%): C; ***CD274***(14%): C
Migration	***CXCR4***(6%): N
Other	***TMEM30A*** (19%): B, C, N; ***FAS*** (18%): C, N; ***NOL9***(18%): B; ***PABPC1***(10%): B; ***DDX3X*** (7%): N; ***PDS5B*** (4%): N

*C1* C cohort, *BCL6-DH* D cohort, *NOTCH2* N cohort, *BN2* B cohort

*Ubiquitin–proteasome system

#### Alterations relating Cellular differentiation and transcription factors

DHL-BCL6 is characterized by the dual translocation of *MYC* and *BCL6*. **BCL6** is a transcriptional repressor, which is vital for the development and maintenance of GC. It regulates complex genetic networks associated with cell differentiation, DNA damage repair, apoptosis, signaling pathways, and T-B cell interactions [144]. Mutations in DHL-BCL6 that coincide with marginal zone lymphomas have been described, and the loss of *KLF2* (a transcription factor involved in B cell homeostasis) increases marginal zone B cell [145].

Mutation of other transcription factors may result in the alteration of intercellular signaling. ETS1, a member of the ETS family namely FLI1, sustain proliferation and survival of B cells [146]. In ABC DLBCL, *ETS1* is overexpressed, resulting in transactivation of genesrelating to BCR and CD40 signaling, NF-κB/TNFα pathways, immune response, and early differentiation. Thus *ETS1* overexpression which promotes B cells growth and proliferation of [147]. *ZEB2* is a transcription factor expressed in various immune cells.Loss of *ZEB2* leads to impaired differentiation and IL-7 signaling, and aberrant proliferation of myeloid cells [148]. Mutation of *POU2F2* is found in both DHL-BCL2 and -BCL6 cohorts [19].

#### Alterations relating to oncogenic pathways

(5) Alterations relating to BCR and NF-κB signaling

Compared to DHL-BCL2, genes associated with BCR and NF-κB pathways are more prominently mutated in the DHL-BCL6 subtype, leading to up-regulated BCR signaling [19–21, 23]. *BCL10* and *PRKCB* encode two components in the BCR and NF-κB signaling transduction [149], which are mutated and amplified in DHL-BCL6 [21, 22]. Inactivating mutations located in *TNFAIP3*, the negative regulator of NF-κB could cause abnormal activation of NF-κB pathway and proliferation of cancer cells [150]. *KLHL21*(CRL3, an E3 ubiquitin ligase) negatively regulating NF-κB by degradation of IKKβ [151] and affecting cell migration [152, 153], is also subjected to heterozygous loss and homozygous deletion in DHL-BCL6 [21, 22].

(6) Alterations relating to the Notch pathway

Once Notch ligand binds to the Notch receptor, the latter will be cleaved by disintegrin, metalloproteinase domain-containing protein (ADAM) at S2 site and γ-secretase at S3 site, to generate Notch extracellular domain, Notch transmembrane domain (NTMD), and Notch intracellular domain (NICD). Afterwards, NICD enters the nucleus, combines with cBF1-suppressor of hairless-LAG1 (cSL) and mastermind-like proteins (MAML1, MAML2, or MAML3), and activates the transcription of related genes [154].

The aberrantly activated Notch pathway, which promotes NF-κB signaling and cell proliferation, usually co-occurs with *BCL6* translocation [19–23]. Notch targeted genes are up-regulated in this cluster, facilitating their differentiation to marginal zone cells[22, 155]. Mutated or truncated Notch2 receptors, as well as inactive mutation of Notch suppressor SPEN, were often found in this cluster, resulting in Notch2 signaling hyperactivation [156, 157]. *DTX1* mutations were also identified, equally in both GCB and non-GCB DLBCLs, and are primarily enriched in the N-terminal protein-interaction domain (WWE1), where the DTX1 binds with Notch and exhibits inhibitory role [158, 159].

#### Alterations relating cell cycle and p53

Cyclin interacts with CDKs to modulate the cell cycle. Mutations located at the 260–290 amino acids of *CCND3* are found both in Burkitt lymphoma and DLBCL, which is likely to increase the stability of cyclin D3, and to further promote cell cycle progression [19, 64, 130, 160]. *CDKN2A* encodes proteins, including p53 stabilizer ARF and p16^INK4a^, which halts cell cycle via antagonizing CDK4/6 to [161]. Recurrent mutations found at R80 may result in the loss of CDK4 inhibiting ability [19, 162].

Besides, p53 pathway can also be affected by the mutated *TRRAP*. *TRRAP* encodes transformation/transcription domain-associated protein (a cofactor of histone acetyltransferases), which also contributes to p53 accumulation [163]. Heterozygous loss of *TRRAP*, therefore, may impair p53 signaling [21, 22].

*TP63* is a member of TP53 family, which encodes two isoforms of p63, including tumor suppressor TAp63 (with N-terminal transactivation domain), and a truncated form ∆Np63 (without N-terminus), possibly an oncoprotein [164]. Mutation, deletion, and *TBL1XR1/TP63* gene fusion may promote oncogenesis via deregulation of p63 and BCL6, BCL2, and MYC [22, 165].

#### Alterations relating to the ubiquitin–proteasome system

The ubiquitin–proteasome system plays a key role in cellular protein degradation. In the system, polyubiquitination of the target protein is mediated sequentially by a single ubiquitin-activating enzyme 1 (E1), multiple ubiquitin-conjugating enzymes (E2), and ubiquitin-protein ligases (E3). Polyubiquitinated protein is later deubiquitinated and degraded to oligopeptides by proteasome complex [166].

Enzymes related to this process are frequently mutated in DHL-BCL6 [19–23]. The mutation of *UBE2A* (ubiquitin-conjugating enzyme E2A) might affect histone H2A ubiquitination and in turn, perturb the transcriptome relating to myeloid development [142, 167]. Ubiquitin E3 ligase *TRIP12* participates in bioprocesses including cell proliferation, DNA repair, and chromatin remodeling via regulating the stability of proteins including PARP1, CDKN2A, RNF168, USP7, and p53. Mutation and increased expression of ubiquitin E3 ligase *TRIP12* are also detected in DHL-BCL6 [168]. As described above, mutation of E3 ubiquitin-protein ligase *DTX1* affects the Notch pathway, while *KLHL21* mutation results in NF-κB upregulation.

#### Alterations relating to immune escape

Surface receptors related to immune response, including CD70, B2M, HLA-B, and CD274, are recurrently mutated in DHL-BCL6 [19–21]. CD70 interacts with CD27 on T cells, mediating T-cell-dependent cytotoxicity and immune escape [169]. CD70 expression is associated with inferior outcomes, while its mutation and deletion are extensively associated with DHL-BCL6 [21, 22, 170]. Perturbed expression of MHC class I complex on cell membrane, resulting from the mutation of its components Beta-2-microglobulin (encoded by *B2M*) and HLA-B, leads to decreased expression of MHC I on the cell membrane and immune escape of B cells from T cells [19, 171, 172]. CD274 or PD-L1 is the ligand of the PD-1 immune checkpoint which is expressed on T cells. Ligand binding triggers a series of downstream cascades, eventually leading to the exhaustion of T cells in the form of decreased proliferation and promoted apoptosis. Recurrent gain and amplification of PD-L1 relate to DHL-BCL6, as well as in non-GCB DLBCL [20, 173]. Besides, a higher level of PD-L1 is often associated with poor prognosis [174].

#### Alterations relating to cell migration

It has been reported that the activation of CXCR4 by its ligand CXCL12 can induce signaling relating to cell migration, survival, and proliferation. In lymphoma, the activation of the CXCL12-CXCR4 axis disseminates B cells into lymph nodes [175] and bone marrow [176] and is correlated with poor survival [177]. It also mediates the immunosuppressive environment in B cell lymphoma through recruitment of regulatory T cells [178]. Besides, the consequence of active CXCR4 signaling in lymphoma also involves oncogenesis [179] and confers resistance to PI3Kδ inhibitor [180], which is disrupted by a missense mutation in DHL-BCL6 [19]. Point mutations at the C-terminal of CXCR4, therefore, may sustain the activation of the CXCL12-CXCR4 axis and contribute to cell dissemination and disease progression [19, 21, 181].

#### Other alterations

Although mutations relating to epigenetics had been found in DHL-BCL6, its scope and frequency are much less than that in DHL-BCL2 [19–21], Lesions affecting histone compartments, as well as epigenetic regulators including *KMT2D* and *ARID1A,* had been identified.

Inactivating lesions targeting *TMEM30A* were identified most frequently in multiple DHL-BCL6 cohorts [19–22]. The *TMEM30A*-knockout model suggested a correlation between *TMEM30A* loss and increased tumor-associated macrophages, up-regulated B cell signaling, as well as better clinical outcomes [182].

## Targeted therapy

The discoveries of genetic lesions of two DHL subtypes identified their distinct biological engagements. With this knowledge, the therapeutic sensitivity or resistance of different DHL subtypes might be inferred, facilitating the exploration of corresponding targeted drugs. Below we summarized novel targeted agents and categorized them based on the heterogeneous genetic alterations in different DHL subtypes. For agents not yet investigated in DHL, we discussed their anti-tumor efficacy in DLBCL and other lymphomas for reference.

### Targeted therapy for DHL-BCL2

The therapeutic vulnerability of DHL-BCL2 lies in its most perturbed proteins, such as MYC, **BCL2**, and epigenetic regulators. However, due to the difficulty in MYC inhibition and lack of efficacy in **BCL2** inhibitors, significant attention was drawn to the application of epigenetic regulating inhibitors in DHL-BCL2, which exhibited satisfactory effect. Inhibition in signaling pathways, such as PI3K-mTOR and JAK-STAT, was also tested in this subtype. The clinical use of these targeted agents is summarized in Table 4.

**Table 4:** Relevant targeted agents in hematological malignances

Category	Target	Agent	Stage of development	References
*MYC* regulators	Myc	CX-3543 (Quarfloxin)	Phase II (B-cell chronic lymphocytic leukemia, withdrawn)	[183]
		INX-3280	Phase II (Terminated)	[184]
		Oncomyc-NG	Phase I (neoplasms, Terminated)	NCT00343148
Apoptosis protein	**BCL2**	Venetoclax	Phase I (NHL)	NCT01328626[185]
	**BCL2** + PI3K	Venetoclax Venetoclax + PI3K inhibitor	Phase I (+ bendamustine and rituximab, r/r NHL)	NCT01594229[186]
			Phase II (+ R-CHOP, DLBCL)	NCT02055820[187]
			Preclinical	[188]
	**BCL2** + SYK/BTK	Venetoclax + SYK/BTK inhibitor	Preclinical	[189]
	**BCL2** + EZH2	Venetoclax + EZH2 inhibitor	Preclinical	[190]
	Mcl-1	PRT1419	Phase I (r/r hematologic malignancies including NHL)	NCT04543305, NCT05107856
		MIK665	Phase I (multiple myeloma, lymphoma)	NCT02992483
		AMG397	Phase I (hematologic malignancies including lymphoma)	NCT03465540
	cIAP1	Xevinapant	Phase I (lymphoma)	NCT01078649
	cIAP1/cIAP2	LCL-161	Phase II (multiple myeloma) Preclinical (r/r lymphoma)	NCT02314052 [191]
	Survivin (BIRC5)	SM1044	Preclinical (DLBCL)	[192]
		YM155	Phase II (lymphoma, B-cell lymphoma, DLBCL, terminated)	NCT00498914
	NMT	PCLX-001	Phase I (lymphoma, NHL)	NCT04836195
Epigenetic regulators	EZH2	Tazemetostat	Phase II (+ R-CHOP for DLBCL; + atezolizumab for DLBCL), Approval for FL	[193–195], NCT02889523, NCT02220842
		Valemetostat	Phase II (adult T-cell leukemia/lymphoma)	[196]
		CPI-1205	Phase I (DLBCL)	[197]
		PF-06821497	Phase I (DLBCL and primary cutaneous follicle center lymphoma)	NCT03460977
		SHR2554	Phase I/II (lymphoma)	NCT03603951
	EED	MAK683	Phase I/II (DLBCL)	NCT02900651
	HDAC (1,2,3,6)	Vorinostat	Phase II (NHL), Approval for CTCL	[198]
	HDAC	Panobinostat	Phase II (+ rituximab for BCL), Approval for multiple myeloma	[199]
		Chidamide	Phase II (DLBCL), Approval for Peripheral T-cell lymphoma	[200]
		Romidepsin	Phase I/II (DLBCL), Approval for T-cell lymphoma	[201]
		Mocetinostat	Phase II (lymphoma)	[202]
		CUDC-907	Phase I (B-cell lymphoma and DLBCL)	[203], NCT02674750
	BRD2-4	OTX015	Phase II (DLBCL)	[204]
	BRD4	CPI-203	preclinical	[205]
		PLX-2853	Phase I (DLBCL and follicular lymphoma)	[206]
		CPI-0610	Phase III (myelofibrosis), Phase I (lymphoma)	[207]
		JQ1	preclinical	[208]
	DNMT1	Azacitidine	+ R-CHOP Phase I/II (DLBCL), Approval for myelodysplastic syndromes, AML and CML	[209], NCT01004991, NCT03450343
		Decitabine	+ R-CHOP Phase I/II (DLBCL), Approval for myelodysplastic syndromes and myelogenous leukemia	NCT02951728
JAK-STAT signaling	STAT3	Danvatirsen (AZD9150)	Phase II (DLBCL)	NCT02983578
	JAK1 and JAK2	Ruxolitinib	Approval for myelofibrosis and lymphoma	NCT00952289 NCT00934544 NCT01243944
	JAK2	Pacritinib	Phase III (myelofibrosis) Phase I (lymphoma)	NCT04404361 NCT01436084 NCT03601819
NF-κB Signaling	IRAK4	KT-474	Phase I (ABC-DLBCL)	NCT04772885
PI3K-AKT-mTOR	PI3K	Idelalisib	Approval for chronic lymphocytic leukemia, Phase II (DLBCL), Phase II (+ Ibrutinib for NHL, withdrawn) Phase I/II (NHL, terminated)	NCT02136511 NCT03576443 NCT02662296 NCT01090414
		Copanlisib	Approval for follicular center lymphoma, Phase III (NHL)	[210] NCT02367040
	AKT	MK-2206	Phase II (DLBCL) Phase II (anaplastic large cell lymphoma)	NCT01481129 NCT01258998
	mTOR	Everolimus	Phase III (DLBCL)	NCT00790036
		Temsirolimus	Approval for NHL, Phase II (DLBCL)	NCT01180049 NCT00290472

#### Targeting MYC

As described previously, *MYC* translocations frequently occur in both types of DHL and are often mutated in DHL-BCL2, as well as other hematological malignancies, making it an attractive target for several malignancies. However, this target has been considered undruggable for a long time since the lack of well-defined ligand binding sites and large protein–protein interaction surfaces [211]. Nevertheless, there were still plenty of efforts focusing on discovering effective molecules that directly target MYC by disrupting the MYC/Max dimerization [212, 213], such as MYCi975 [214], 10074-G5 [215], and JY-3-094 [216], or by interfering the formation of c-MYC/Max/DNA complex, like MYRA-A [217] and NSC308848 [218]. Unfortunately, none of them has been promoted to clinic evaluation mainly due to the low efficacy and/or limited in vivo tolerability. Given such a situation, novel medicinal chemistry-based pharmacophore discovery and optimization, or induced-proximity approaches with catalytical mechanisms, such as PROTAC, could be employed to improve the efficacy and broaden the therapeutic window.

Instead of direct strategy, targeting *MYC* gene transcription or translation is an effective strategy. G-quadruplexes (also known as G4-DNA) are a tertiary structure formed by guanine-rich sequences in nucleic acids. CX-3543 (Quarfloxin) was the first G-quadruplex stabilizer entering clinical trials. It was recently found to down-regulate MYC by damaging the function of G-quadruplex binding nucleolar proteins and *MYC* transcription [213, 219]. Oligonucleotide and siRNA technology have also been applied to inhibit *MYC* gene expression [211]. OmoMYC, as a small protein containing the bHLH-zip domain of MYC, is another MYC family inhibitor [211]. OmoMYC inhibits the binding of c-MYC, *n*-MYC, or l-MYC to Max and prevents MYC-Max heterodimer from interacting with E-box [220, 221]. Recently, a group of natural products (Rocaglates) and their synthetic derivatives have been emerging as promising therapeutical agents for the treatment of MYC-associated lymphoma, especially those Double Hit lymphoma with concurrent MYC and BCL2 dysregulation [222, 223]. These molecules can efficiently inhibit MYC expression and tumor cell viability by stabilizing target mRNA–eIF4A interaction that directly prevents translation. In addition, PLK1 was reported as key factor on sustaining MYC activity through GSK3β-mediated MYC protein stability in DHL. Therefore, inhibition of PLK1 with small molecules, such as volasertib and Ro3280, can also downregulate the protein level of MYC [224].

Although no inhibitor of MYC is effective in DHL, given its specificity, these inhibitors are expected to be the potential therapy for DHL. In addition, MYC function can be affected by BET inhibitors via transcription and destabilization of MYC protein, which leads to the development of BET inhibitors, including JQ1, and will be further discussed in the section of epigenetic regulators [225–227].

#### Targeting apoptosis

The anti-apoptotic nature and up-regulated level of **BCL2** in the majority of DHL-BCL2 lymphoma and other malignancies have raised interest in developing inhibitors targeting **BCL2** and its family members. **BCL2** inhibitors mimic BH3-only proteins which bind to the pro-survival members of the **BCL2** family and thus trigger apoptosis.

**Fig. 3:**
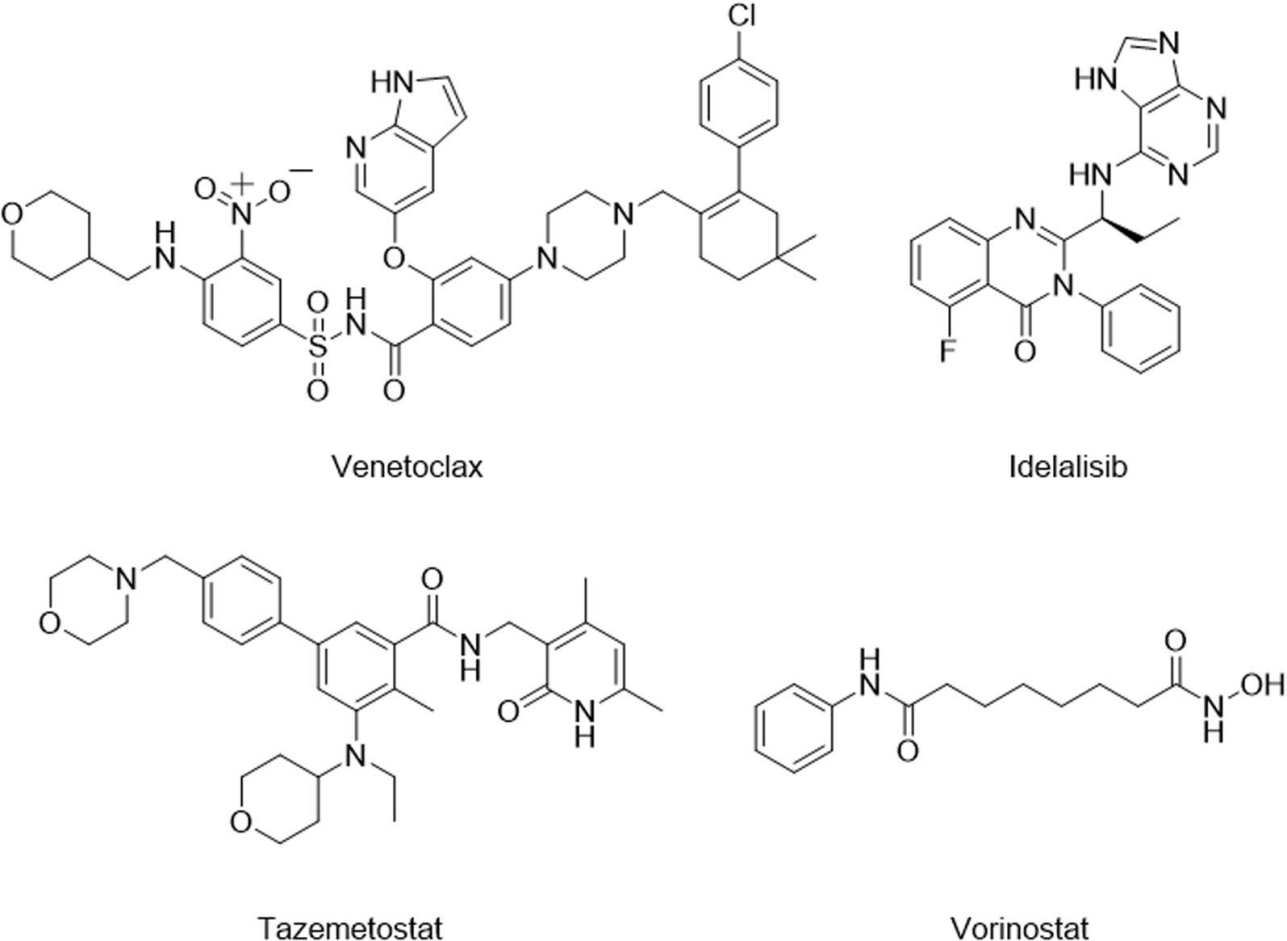
Structures of targeted agents in DHL-BCL2

Venetoclax, a **BCL2** inhibitor with good efficiency in leukemia, was observed with limited activity in DLBCL patients with ORR only at 18% [185], and is now being tested as a regimen in several combination therapies including chemotherapy [228], radiotherapy [229] and targeted therapy, such as PI3K, BTK and SYK inhibitors (Fig. 3) [185, 188, 230]. In the CAVALLI phase, 2 studies of venetoclax plus R-CHOP as first-line treatment of DLBCL, the 2-year PFS and OS of venetoclax plus R-CHOP were higher than R-CHOP alone, observed in all DLBCL patients (2-year PFS: 80% vs 67%; 2-year OS: 86% vs 81%), as well as patients with **BCL2** + DLBCLs and DELs. AEs were also increased in venetoclax plus the R-CHOP group but were considered manageable [187]. In a preclinical study, the combination of copanlisib (PI3K inhibitor) and venetoclax extended the median survival of the DLBCL mice model bearing BCL2 translocation and DHL-BCL2-like genetic dysregulations [188]. This synergistic effect may contribute to the inhibited PTEN and enhanced Akt pathway function during venetoclax administration [231], which partially explains the similar effect observed in the study of SYK/BTK inhibitor and venetoclax combination [189]. Besides the inhibition of the Akt pathway, inhibitors of EZH2 were also found to sensitize IGH/BCL2 translocated DLBCL cells [190]. In all, the use of **BCL2** inhibitor alone may fail to achieve a satisfactory effect in lymphoma, but the combined use of **BCL2** inhibitor and other DHL-BCL2 targeted drugs is promising, especially in **BCL2** aberrant DLBCLs.

Other anti-apoptosis proteins in the BCL2 family, like BCL-XL and MCL-1, have also been used in the treatment of other hematological malignancies. Among them, MCL-1 inhibitor has been tested in several clinical trials for the treatment of DLBCLs. Additionally, researches indicate that the upregulation of BCL-XL and MCL-1 may be an underlying mechanism in the BCL2 inhibitors resistance in hematological carcinoma [232, 233]. Also, dual inhibition of BCL2 and MCL-1 on DLBCL exhibits synergic effects both in vitro and in vivo [234, 235]. Therefore, MCL-1 is considered a potential target for DLBCL, and MCL-1 inhibition may be curative for patients with Venetoclax resistance.

AMG 176, an MCL-1 inhibitor that exhibited anti-tumor activity against DLBCL and other hematological malignances in vitro [236], has entered the Phase I study in combination with Venetoclax (NCT03797261). However, the trial is terminated due to cardiac toxicity. Other MCL-1 inhibitors, including PRT1419 (NCT04543305, NCT05107856), MIK665 (as single drug: NCT02992483, terminated, in combination with a BCL2 inhibitor, VOB560: NCT04702425) and AMG 397 (NCT03465540), had also been evaluated in Phase I studies for the treatment of hematological carcinoma including DLBCL. Most results are yet unavailable, while the trial on AMG 397 is currently on voluntary hold.

In addition to the BCL2 family, inhibitors of apoptosis (IAPs) are other sets of anti-apoptotic protein, which are mainly divided into three categories: IAP1 proteins (cIAP1, BIRC2), IAP2 proteins (cIAP2, BIRC3), and XIAP proteins (XIAP, BIRC4). These proteins are essential for activating downstream signaling of the NF-κB pathway, producing pro-survival transcriptional signals [237]. Mitochondrial protein SMAC is an endogenous inhibitor of IAPs which combines and antagonizes IAPs, subsequently activating caspase-3/7/9 and promoting apoptosis [238]. SMAC mimics are used as antagonists of IAPs, and some have entered the clinical trials as a single agent, combination therapies, and immunomodulatory agents [239, 240].

Survivin is a highly conserved member of the apoptotic protein (IAPs) inhibitor family which is overexpressed in up to 60% of DLBCL cases [241]. Therefore, some Survivin inhibitors were tested on DLBCL models. Survivin inhibitor sepantronium Bromide (YM155) entered the clinical study for NHL including DLBCL, yet was terminated in phase II because of insufficient efficacy (NCT00498914) [242]. Combination therapy of YM155 with rituximab therapy was also explored. The combined treatment of YM155 and rituximab enhances the antitumor activity of B-NHL xenografts. Compared with monotherapy, the combined treatment extends the survival time of severely combined immunocompromised mice with WSU-FSCCL and Jeko B-NHL diffuse tumors. Good antitumor activity and low toxic side effects are shown in phase II [243]. When combined with bendamustine and rituximab, stronger tumor lethality and lower toxic side effects were observed, which can decrease FLT-PET signal in lymph nodes and prolong overall survival in mice bearing disseminated DLBCL xenografts, and perhaps relapsed/refractory large B-cell lymphoma as well [244]. Another survivin inhibitor SM1044 is a new water-soluble artemisinin derivative of antimalarial drug, inducing the degradation of survivin through acetylation-dependent interaction with LC3-II to promote the apoptosis of the DLBCL cell line. It also activates the CaMKK2-AMPK-ULK1 axis, which leads to the initiation of autophagy [192].

Inhibitors of other IAP proteins are also under clinical evaluation. Xevinapant is an inhibitor of cIAP protein and is currently in clinical phase I for the treatment of lymphoma. Preclinical evaluations have shown that it has good pharmacokinetic properties in animal models [245] and the dose of less than 400 mg/day combined with daunorubicin and cytarabine can reduce drug resistance during the treatment of acute myeloid leukemia [246]. IAP inhibitor LCL-161 showed therapeutic effects in cell models of rituximab-resistant B-cell lymphoma, suggesting a potential to treat DLBCL patients previously treated with rituximab [191]. LCL161 is currently in clinical trials for a number of hematological malignancies, including B-cell lymphoma [247].

There are also apoptosis inducers, such as PCLX-001. It is an N-myristoyltransferase (NMT) inhibitor, which is currently in Phase I and is mainly used in the treatment of NHL. Treatment of PCLX-001 impacts the global myristoylation of lymphoma cell proteins and inhibits early BCR signaling, which is critical for survival [248]. In addition, PCLX-001 has a high selectivity and significantly promotes the production of numerous non-myristoylated BCR effectors, including c-MYC, NF-κB, and p-ERK. It can be used as a precision drug, with researchers predicting minimal side effects during treatment [249].

#### Targeting epigenetic regulation

1. Targeted histone methylation therapy

*KMT2D* mutation leads to a significant decrease in H3K4 monomethylation in tumor cells, an increase of genomic DNA damage and mutation load, as well as transcriptional instability [250]. As mentioned above, lysine-specific histone methyltransferase *KMT2D* is one of the most frequently altered genes in DHL-BCL2 [23]. Although few drugs have been reported against *KMT2D*, novel histone demethylase inhibitors might solve the loss of methylation typically observed in *KMT2D*-inactivated tumors [251]. In addition, the *KMT2D* mutation in DHL causes DNA damage and increased transcriptional stress, resulting in the accumulation of a higher mutation load and the generation of more tumor neoantigens [252]. These changes lead to a higher level of immune cell infiltration in the tumor microenvironment, making it more sensitive to immune therapy.

Mutations in EZH2 are also prevalent in DHL patients, suggesting that EZH2 inhibitors may be a potential targeted drug for the treatment of DHL. Tazemetostat is an oral selective potent EZH2 inhibitor. It was firstly approved for clinical trials in 2013. In the phase, I clinical trial, tazemetostat therapy has demonstrated preliminary efficacy in 38% of patients with relapsed/refractory B-cell lymphoma, 14% with CRR, and median progression-free survival (mPFS) of 12.4 months. Grade 3 and above adverse reactions were mainly thrombocytopenia (14%) and neutropenia (14%) [193]. The interim results of phase II clinical trial showed a significant increase in ORR in DLBCL and follicular lymphoma patients with EZH2 mutant compared with wild-type (40% vs. 18%;63% vs. 28%) [194, 195]. In addition, the Phase II clinical trial of tazemetostat combined with R-CHOP (NCT02889523) and PD-L1 antibody atezolizumab (NCT02220842) in the treatment of DLBCL are in progress.

Valemetostat, as a dual inhibitor of EZH 1/2, has shown more potent antitumor efficacy than EZH2 inhibitors [196]. In phase, I a clinical trial with 15 patients with NHL patients treated by Valemetostat showed the ORR of 53% and CBR of 86%. The ORR of patients with peripheral T-cell lymphoma reached 80%, indicating the reliable efficacy of valemetostat in the treatment of NHL. CPI-1205 is a novel oral small molecule EZH2 inhibitor, which achieves demethylation by competitively inhibiting the binding of SAM to the EZH2 catalytic group. Harb et al.[197] found that, among the 28 BCL patients treated with 6 cycles of CPI-1205, one case achieved CR and five cases achieved stable disease (SD). In addition, next-generation EZH2 inhibitors, including CPI169, CPI360, MAK683, PF-06821497, and SHR2554, are currently recruiting lymphoma patients for clinical trials (NCT02900651, NCT03460977, NCT03603951).

(2) Targeted histone acetylation therapy

Loss-of-function mutations in genes encoding proteins with a defined role in histone acetylation (e.g., *CREBBP* or *EP300*) are commonly observed in DHL-BCL2 patients (more than 50%). Somatic mutations in *CREBBP* lead to impaired p53 activation and also promote the oncogenic effects of BCL6 [253]. Loss-of-function mutations in *CREBBP* also lead to the silencing of some genes involved in MHC-II-mediated antigen-presentation [42, 96], suggesting that it may promote the immune escape of tumor cells. Taken together, these findings indicate that the regulation of histone acetylation is a potential vulnerability of DHL targeted therapy.

Vorinostat is an oral histone deacetylase inhibitor (HDACi), which works by inducing histone acetylation to activate the expression of cell cycle factors and tumor suppressor genes. In 2014, researchers began exploring the use of Vorinostat in B-cell lymphoma (Fig. 3). In phase II clinical trial involving 39 patients with relapsed/refractory follicular lymphoma [198], the ORR was 49% and mPFS was 20 months. The major adverse reactions were thrombocytopenia and neutropenia. These results suggest that Vorinostat can provide sustained clinical benefit for B-cell lymphoma patients with controllable adverse reactions.

Panobinostat was the first HDAC inhibitor for the treatment of multiple myeloma, and in recent years it has also been explored for B-cell lymphoma. The efficacy of panobinostat in combination with rituximab was reported in over 40 patients with relapsed/refractory DLBCL, with an ORR of 28%, CRR of 18%, and MPFS of 14.5 months [199].

Chidamide, a selective HDAC inhibitor, has been shown to have a synergistic effect with rituximab in the treatment of DLBCL in vitro and in vivo [200]. The loss of CD20 on the cell surface is the main difficulty in the treatment of relapsed/refractory DLBCL with rituximab. Chidamide significantly enhanced the expression of CD20 on the surface of DLBCL cells, thus synergizing with rituximab to exert anti-tumor effects [254]. In DLBCL xenograft mice, chidamide and rituximab synergically induce cell death and inhibited tumor growth [200].

Batlevi et al. completed a phase II clinical trial to evaluate the effect of mocetinostat in FL and DLBCL, and the ORR and clinical benefit rate (CBR) in DLBCL patients were 18.9% and 54.1%, respectively [202]. Romidepsin, an approved macrocyclic pan-HDAC inhibitor, was shown to exert synergistic antitumor effects with GSK126, an EZH2 inhibitor, in the SU-DHL-10 xenograft model [201]. Notably, the novel dual HDAC and PI3K inhibitor CUDC-907 demonstrated excellent activity (ORR 55%) and tolerability (43% of grade ≥ 3 adverse events) in a phase I trial in patients with DLBCL, FL, and HL [203]. CUDC-907 is currently in phase II clinical trial in patients with DLBCL (NCT02674750).

(3) Targeted bromodomain and extra-terminal domain family therapy

The bromodomain and extra-terminal domain (BET) family consists of four proteins: BRD2, BRD3, BRD4, and BRDT, while its N-terminal contains two BRD modules involved in acetyllysine recognition. BRD4 is presented in several transcriptional complexes, including the cofactor regulator and p-TEFB extension factor [255]. The C-terminal domain also regulates the interactions between BRD4 and many well-known transcriptional regulators, notably p-TEFB, MYC, NF-κB, and p53 [255–257]. Although BET mutations or translocations are rare, BET may be overexpressed [258]. Therefore, BET inhibition has been effective in preclinical studies of multiple cancer types, especially for many hematopoietic system cancers that rely on constant BRD4 activity to express MYC [259–261].

OTX015 is the first BET inhibitor (BETi) to reach clinical trials. Amorim et al. [204] conducted a phase I clinical trial in 17 DLBCL patients treated with OTX015, which showed a CBR of 47%, suggesting that OTX015 may have potential antitumor activity against lymphoma. The most common grade 3/4 ADRs included thrombocytopenia (58%), anemia (27%), and neutropenia (22%). Further studies showed that OTX015 can inhibit TLR/NF-κB and JAK/STAT signaling pathways by down-regulating MYC expression and play a targeted therapeutic role [259]. In DHL/THL model, OTX015 (Birabresib) combined with BCL2 antagonist (such as Venetoclax) synergically inhibited the malignant proliferation of DHL/THL cells [262], supporting the combining use of BETi and BCL2 inhibitors against MYC-driven lymphoma. A recent study showed that nonbenzodiazepine BETi, PLX-2853, synergize with venetoclax to induce apoptosis in MYC-driven lymphomas with high BCL2 expression [206]. Similarly, in DHL patients, resistance to venetoclax was overcome by the use of the BET inhibitor CPI-203, possibly due to the down-regulation of BCL2-like protein (BFL1) [205]. In phase I clinical trial with 44 patients with relapsed/refractory lymphoma (DLBCL, FL, and HL) enrolled, 12 patients achieved a moderate response, and 2 patients with DLBCL achieved CR after 5 and 6 cycles of CPI-0610 (a highly selective BETi) treatment, respectively [207]. The treatment of JQ1 (a classic inhibitor of BRD4) extended survival in a mouse xenograft model of MYC-driven lymphoma [208], suggesting that BETi has broad therapeutic potential in DHL that is highly associated with MYC.

(4) Targeted DNA methylation therapy

DNA methylation is one of the most widely studied epigenetic modifications [251]. DNA methylation refers to the formation of 5-methylcytosine by adding methyl at the 5' end of cytosine nucleotide of C-phosphodiester-G (CpG) under the catalysis of DNA methyltransferase (DNMT). A conformational change in the methylated CpG sequence prevents transcription factors from binding, thus silencing the expression of methylated genes [263]. In normal cells, CpG islands are usually in a non-methylated or hypomethylated state, and hypermethylation will inhibit the expression of genes. Some studies suggested that high expression of DNMT3b may be associated with a poor prognosis of DLBCL. The FDA-approved DNMT inhibitors, 5-azacitidine, and decitabine, effectively demethylate DNA and induce the expression of related tumor suppressor genes [251]. In phase I clinical trial [209] involving 33 patients with DLBCL and high-grade follicular lymphoma, 5-azacitidine combined with R-CHOP regimen showed a higher objective response rate (ORR), complete response rate (CRR), and lower incidence of ADRs than R-CHOP.

#### Targeting oncogenic pathways

1. Targeting JAK-STAT Signaling

The JAK/STAT pathway is often abnormally activated in patients with lymphoma, especially in DHL-BCL2, making it a promising target [119]. Disrupted or dysregulated JAK-STAT functionality could result in an altered microenvironment to allow for immune evasion of tumor cells [264]. STAT3 expression and activation are significantly higher in ABC DLBCL cell lines and these cell lines demonstrate higher NF-κB activity than those with low STAT3 activity [265]. Knockdown of *STAT3* in mouse xenografts models suppressed the growth of ABC DLBCL tumors, validating STAT3 as a therapeutic target in this subtype of DLBCL. Due to the difficulties in developing inhibitory molecules for STAT3, plenty of effort was put into the downregulation of STAT3.

SD-36, a novel selective STAT3 degrader, can significantly and selectively degrade STAT3 in different cell lines [266]. According to preclinical data, SD-36 had a significant inhibitory effect on the growth of five DLBCL cell lines. What’s more, SD-36 effectively inhibited tumor growth at 100 mg/kg in the SU-DHL-1 xenograft model and had a complete and long-lasting PD effect in inducing STAT3 degradation in vivo [267].

Danvatirsen (AZD9150) is an antisense oligonucleotide designed to reduce the production of STAT3. Depletion of STAT3 with AXD9150 in KARPAS-299 and SUP-M2 cells and xenograft models rapidly induced apoptosis and reduced the expression of STAT3 pro-survival targets, including MCL-1, c-MYC, BCL6, CYCLIN D1, BIRC5 (SURVIVIN), and IL2Rα [268]. These findings led to a phase I dose-escalation study of AZD9150 in patients with advanced cancer, including 12 lymphoma patients, 7 of them with DLBCL. Anti-tumor activity was shown in these heavily pretreated patients [269, 270]. It is currently used as the treatment of solid tumors and lymphomas (including DLBCL) that have relapsed or are ineffective against multiple chemotherapy regimens.

The targeting inhibition of STAT3 also can be achieved by inhibition of its upstream kinase including JAK1 and JAK2. Ruxolitinib, a non-selective JAK1 and 2 inhibitor that blocks phosphorylation of STAT1 and STAT3, has been approved for primary myelofibrosis [271]. Phase I/II studies evaluating this agent either as monotherapy or in combination with bortezomib in relapsed/refractory DLBCL are ongoing [272]. Pacritinib (formerly SB1518) is an oral small molecule that selectively and potently inhibits JAK2, showing promising in vitro activity in JAK2-dependent DLBCL cell lines independent of JAK2 mutational status [273]. In a phase I study in 34 patients with relapsed or refractory lymphomas, including DLBCL, pacritinib demonstrated a favorable toxicity profile [274].

(2) Targeting PI3K-AKT-mTOR signaling pathway

As previously mentioned, the PI3K/AKT/mTOR pathway was proved to play an essential role in the development and progression of many hematological malignancies, including DLBCL [275]. Inhibitors of this pathway are mainly composed of PI3K, Akt, and mTOR inhibitors.

There are many PI3K inhibitors currently in the market or clinical trials stage for the treatment of hematological tumors. Idelalisib has demonstrated antitumor activity in indolent B-NHL (iB-NHL) [276] but it is less effective in the treatment of DLBCL as a single agent regimen (Fig. 3). When combined with anti-CD20 monoclonal antibodies or BTK inhibitors, anti-tumor efficacy could be. Through complementary mechanisms, combined use with BTK inhibitors can enhance the sensitivity of DLBCL cells, overcome drug resistance, and enhance tumor lethality [277]. So far, some combination regimens have reached clinical trials [278].

Copanlisib was approved for the treatment of follicular center lymphoma [210]. Preclinical experiments have shown that Copanlisib exhibits cell-type-specific cytotoxicity at nanomolar concentrations against DLBCL cell lines. A phase II clinical trial showed that the ORR of all types of DLBCL is 25%, and the ORR for ABC-DLBCL is 38%[279].

Enzastaurin is currently in phase III. It can delay the deterioration of DLBCL despite its poor treatment effect, and have low toxicity and side effects [280]. The first result about enzastaurin for relapsed/refractory DLBCL patients in Phase II was published in 2007 [281]. it showed that none of the disease-related parameters were correlated with response to enzastaurin. Despite the low response rate, the long-term response and the good safety in Phase II warrant further evaluation of enzastaurin together with R-CHOP therapy. Besides, a synergistic effect of enzastaurin with bortezomib and gemcitabine was found in a xenograft model of GCB-DLBCL [282].

The AKT inhibitor MK-2206 is in phase II and is mainly used for the treatment of non-small cell lung cancer, colorectal cancer, while also exhibiting a therapeutic effect on DLBCL. The combination of MK-2206 and mTOR inhibitor nelfinavir can overcome the drug resistance of mTOR inhibitors in DLBCL, reduce the viability of DLBCL cells and halt the cell cycle, promoting cell apoptosis [283]. MK-2206 monotherapy kills lymphoma cells by reducing the level of p-AKT, inhibiting the downstream targets of AKT signaling, and inducing Rictor and phosphatidylinositol 3-kinase expression. The sensitivity of MK-2206 is related to the activation state of AKT in DLBCL cells. Although monomer therapy is also effective, AKT inhibitors are combined with other targeted drugs for better clinical efficacy [284].

Everolimus is an mTOR inhibitor that was approved in 2003 to treat breast cancer and kidney disease. Its research for DLBCL is in phase III studies which showed that oral Everolimus can induce BLDCL cell cycle arrest. According to the result of the Phase I study, the best dose of Everolimus is 10 mg/d [285]. Results from the phase II study showed the ORR of Everolimus DLBCL is 30%. Meanwhile, Everolimus combined with rituximab hassled to stronger cell lethality. The objective response rate was 38%, the complete response rate was 13%, and there was no increase in toxicity [286].

Temsirolimus is also an mTOR inhibitor approved for the treatment of NHL in 2007 [287]. It is in phase II for DLBCL. It can inhibit the growth of GCB-DLBCL and ABC-BLDCL cells (GCB = 30–66%, ABC = 45–57%). Combination therapy enhances its therapeutic effect, and combined with different drugs can be used to treat different types of DLBCL. For example, its combination with idelalisib enhanced the lethality of GCB-DLBCL and ABC-BLDCL cells, while combining with ibrutinib or bortezomib, a better therapeutic effect on ABC-DLBCL or GCB-DLBCL was observed, respectively [288].

#### Other therapies

Since DHL-BCL2 is accompanied by a high frequency of *TNFRSF14* and *ARID1A* mutation, certain unique vulnerabilities were found in these subsets of tumors.

As mentioned above, *TNFRSF14* mutation brings about reduced HVEM levels, resulting in BTLA-mediated proliferation and CD40 signaling. Stimulation of HVEM by human LIGHT, its ligand, renders NHL more immunogenetic and sensitive to Fas-induced apoptosis, without inducing proliferation [135]. Also, restoration of HEVM ectodomain by soluble HVEM protein or HVEM-producing and CD19-targeted CAR-T cells were tumor-suppressive in MYC + /BCL2 + DLBCL cell lines and BCL2 overexpressing lymphoma xenograft model, respectively [134]. These results indicated the breakthrough point for the intervention of immunotherapy in DHL-BCL2.

*ARID1A* and its homolog *ARID1B* have a synthetic lethal relationship. In *ARID1A* mutated cancers, the co-mutation of *ARID1B* destabilizes the SWI/SNF complex and damages cell proliferation, suggesting a potential target for malignancies harboring *ARID1A* mutations [107]. Also, researchers found that tumors with *ARID1A* deficiency were sensitive to immune checkpoint blockade (targeting PD-1–PD-L1), bringing a novel perspective for treating DHL-BCL2 [289].

### Targeted therapy for DHL-MYC/BCL6

Compared to DHL-BCL2, targeted agents for DHL-BCL6 were less subtype-specific due to insufficient understanding of its biology and diverse cell-of-origin. Here, we selected drugs targeting genetically disturbed proteins or pathways found in DHL-BCL6, hopefully providing insight into the treatment of DHL-BCL6. Clinical tests of these inhibitors are summarized in Table 5.

**Table 5:** Targeted therapy for DHL-BCL6

Category	Target	Agent	Stage of development	References
Notch	γ-secretase	MK-0752	Phase I (CLL, terminated)	NCT00100152
		Z-LLNle-CHO	Preclinical (precursor B-cell acute leukemia)	[290]
		Z-IL-CHO	Preclinical (DLBCL)	[291]
	Unidentified	Crenigacestat	Phase I /II (multiple myeloma and precursor T-cell lymphoblastic leukemia)	NCT04855136 NCT02518113
	Unidentified	CB-103	Phase I (NHL)	NCT03422679
BCR signaling	BTK	Ibrutinib	Approval for mantle cell lymphoma, Chronic lymphocytic leukemia/small lymphocytic lymphoma, DLBCL	NCT01236391 NCT01105247 NCT01578707
	SYK	Fostamatinib Disodium (R788)	Approval for DLBCL	NCT02076399 NCT02076412 NCT02077192
NF-κB signaling	IRAK4	KT-474	Phase I (ABC-DLBCL)	NCT04772885
Ubiquitin proteasome system	CRBN	Lenalidomide	Phase II (+ DA-EPOCH-R, lymphoma including DLBCL)	NCT02213913 [292]
			Phase II (+ R-CHOP, lymphoma including DLBCL)	NCT00670358 [293]
			Phase II (+ R-CHOP21, lymphoma including DLBCL)	NCT00907348 [294]
			Phase II (+ rituximab, lymphoma including DLBCL)	NCT00294632 [295]
			Phase II (+ ibrutinib and rituximab, r/r DLBCL)	NCT02077166 [296]
			Phase III (maintenance regiment, DLBCL)	NCT01122472 [297]
	Proteasome	Bortezomib	Approval for multiple myeloma, Phase III (NHL and DLBCL)	NCT02268890 NCT00312845 NCT01324596
		ixazomib	Approval for multiple myeloma, Phase I/II (DLBCL)	NCT03173092 NCT02481310
	Proteasomal USP14 and UCHL5 deubiquitinases	b-AP15	Preclinical (DLBCL)	[298]
p53 signaling	p53	Eprenetapopt (APR-246)	Phase II (Leukemia)	NCT03588078
	MDM2	Idasanutlin (RG7388)	Phase III (+ Cytarabine, leukemia)	NCT02545283
XPO1	XPO1	Selinexor (KPT-330)	Approval for r/r DLBCL and r/r multiple myeloma	[299]
Proliferation	CDK4/6	Palbociclib	Phase II (+ Ibrutinib for mantle cell lymphoma and B-cell lymphoma)	NCT03478514
		Abemaciclib	Phase II (MCL)	NCT01739309
	CDK9	Dinaciclib	Phase Ib (+ Pembrolizumab for DLBCL)	NCT02684617 (Terminated); [234]
		Voruciclib	Phase I (DLBCL)	NCT03547115; [300]
CXCR4	CXCR4	PF-06747143	Preclinical	[301]
	CXCR4-directed auristatin E nanoconjugate	T22-AUR	Preclinical	[302]
	CXCR4 modified	CD19 CAR T cells	Phase I (refractory NHL)	NCT04684472
	CXCR4-directed	radioligand therapy	Preclinical	[303]
Immunotherapy	PD-1	Nivolumab	Phase 1b (r/r DLBCL)	NCT01592370 [304]
			Phase II (DLBCL, patients with relapse after or were ineligible for autologous hematopoietic cell transplantation)	NCT02038933 [305]
		Pembrolizumab	Phase 1 (hematology malignancies including DLBCL)	NCT01953692 [306]
			Preclinical (+ R-CHOP, in R-CHOP untreated DLBCL)	[307]

#### Targeting oncogenic pathways

1. Targeting Notch pathway

Despite the frequent activation of the Notch pathway in DHL-BCL6, drugs targeting this cascade were rarely tested in DHLs, even DLBCLs. Inhibition of the γ-secretase is a common strategy in targeting the Notch pathway, which is mainly used in solid tumors [308], with a few were tested in hematology malignancies in pre-clinical studies. Z-IL-CHO (GSI-XII) can reduce the mRNA content of HES1 in TMD8 cell lines established from DLBCL patients, proving its potential therapeutic effect in DLBCL [291]. A combination of Z-IL-CHO and bortezomib can enhance the cytotoxicity of bortezomib in multiple myeloma. This process is mainly caused by the synergistic inhibition of chymotrypsin-like proteasome by Z-IL-CHO and bortezomib, rather than the inhibition of the Notch pathway, which provides a choice for the combined treatment of multiple myeloma [309]. Z-LLNle-CHO (GSI-I), crenigacestat, and MK-0752 all exhibited inhibitory activity against γ-secretase and were also tested in different hematology malignancies, yet no studies in DLBCL were launched [290, 310–312].

Another molecule, CB-103, acts as a disruptor of NICD protein–protein interaction and inhibitor of the active forms of Notch receptors. It targets various cancers with Notch overexpression resistant to Notch inhibition by GSIs and monoclonal antibodies while eliminating the gastrointestinal toxicity and expanding the therapeutic window of the first—and second-generation Notch inhibitors [313, 314]. Likely, evaluation of these inhibitors may yield unexpected results in the DHL-BCL6 subtype.

(2) Targeting BCR Signaling

The BCR complex and associated protein tyrosine kinases are essential for normal B-cell function and antibody production. BCR cross-linking activates 3 main pathways: BTK, PLC-γ2, and PI3K [315]. Constitutively activated BCR signaling is linked to the initiation and maintenance of B-cell malignancies, especially in the ABC-DLBCL. Drugs targeting these proteins either as adjuncts to R-CHOP in the frontline setting or as monotherapy/combination therapy in the relapsed/refractory setting are under evaluation [316].

**Fig. 4:**
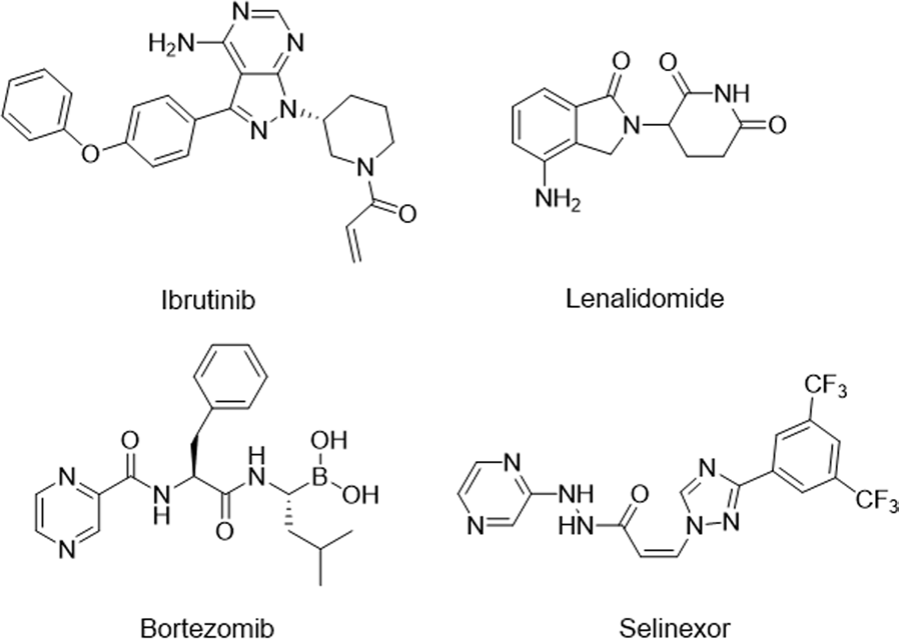
Structures of targeted agents in DHL-BCL6

Ibrutinib, a first-in-class small molecule that selectively covalently binds to the cysteine residue (Cys-481) of the active site of BTK, irreversibly inhibits its activity (Fig. 4) [317]. For patients with ABC DLBCL, the effective rate of single ibrutinib in relapsed/refractory patients can be as high as 37%, but for patients with GCB DLBCL, the effective rate is only 15%. In terms of combination therapy, the combination of ibrutinib and R-CHOP can significantly improve the efficacy of R/R DLBCL, and the effectiveness of the GCB subtype is still inferior to that of the ABC subtype [318].

Fostamatinib Disodium (R788) is a prodrug of the active metabolite R406, which is an oral SYK inhibitor with IC50 of 41 nM. It strongly inhibits SYK but does not inhibit Lyn, while the effect on Flt3 is 5 times weaker [319]. However, the results of two pivotal phases III clinical trials OSKIRA-2 and OSKIRA-3 showed that compared with the placebo group, fostamatinib did not show significant clinical benefit in ABC DLBCL [320]. At the same time, phase II clinical studies of hematological malignancies did not show positive results.

Cerdulatinib (PRT-062070) is an orally active, multi-targeted tyrosine kinase inhibitor with IC50 of 12 nM/6 nM/8 nM/0.5 nM and 32 nM for JAK1/JAK2/JAK3/TYK2 and SYK, respectively [321]. It has broad anti-tumor activity in both ABC and GC cell lines of diffuse large B cells at least in part by inhibiting SYK and JAK pathways [322].

Enzastaurin, an oral PKC-β agent, showed limited cytotoxicity in clinical phase I trials and achieved encouraging clinical effects in the phase II trial of DLBCL as induction therapy [323]. However, the PRELUDE randomized phase III trial of R-CHOP ± enzastaurin did not improve PFS as a primary endpoint.

(3) Targeting NF-κB Signaling

The excessive activation of NF-κB dependent gene expression is an important characteristic of the more aggressive ABC DLBCL subtype [324]. The formation of the CARD11/ BCL10/MALTI complex activates IKK, causing phosphorylation and breakdown of IκB, and ultimately leading to the activation of NF-κB [325]. In addition, the adaptor protein MYD88 acts as a mediator, which can accept Toll-like receptor signals to activate NF-κB, and MYD88 is highly mutated in ABC DLBCL [326]. The NF-κB pathway as a therapeutic target was first demonstrated by the IκB kinase (IKK) complex inhibitors in selective inhibition of ABC DLBCL cell lines. Moreover, proteasome inhibition has been shown particular efficacy in patients with ABC DLBCL.

KT-474, a novel heterobifunctional IRAK4 degrader that targets both IRAK4 and IMiD biology leads to potent cell kill in ABC DLBCL lines harboring MYD88 L265P through down modulating survival signals, including NF-κB and autocrine IL-6/IL-10 engagement of the JAK-STAT3 pathway. KT-474 induced superior cellular toxicity compared to IRAK4 kinase inhibition as determined by lower IC50 and induction of apoptosis. MYD88-mutated ABC-DLBCL cell lines are more sensitive to KT-474 exposure as compared to wild-type. A combination of KT-474 in conjunction with ibrutinib, venetoclax and umbralisib shows a synergistic effect in the OCI-LY10 cell line.

#### Targeting ubiquitin–proteasome system

As many pathways are interfered with by genetic alteration, remodeling intercellular signaling via the inhibition of ubiquitin–proteasome-dependent degradation has been considered as a possible strategy, since several important proteins, including IκB, the inhibitory switch of NF-κB, p53, a tumor suppressor, and other ubiquitin-conjugating enzymes found to be mutated in the DHL-BCL6 are associated with ubiquitin–proteasome system. Although no cogent evidence supports the specialty of ubiquitin–proteasome regulator in this subtype, inhibitors regulating the NF-κB pathway exhibited cytotoxicity selectively towards ABC-DLBCL [327], which is more prominent in the DHL-BCL6.

Lenalidomide targets the E3 ubiquitin ligase component cereblon that modulates the interferon regulatory factor 4 and down-regulates the NF-κB pathway in ABC-DLBCL (Fig. 4) [328]. In clinical studies, lenalidomide exhibited promising efficacy in DLBCLs, especially in non-GCB subtype, with significantly prolonged PFS and OS, either as a single agent [293] or in combination with chemotherapies (R-CHOP and similar therapies) [293, 294] or other regiments(rituximab or ibrutinib) [295, 296], as well as being maintenance strategy [297]. For DHL, the response to lenalidomide plus DA-EPOCH-R is less satisfied compared to DELs in terms of disease remission, PFS, and CNS involvement [292]. The difference may be attributed to the small DHL patient group or the higher correlation between DEL and non-GCB lymphoma, which respond better to lenalidomide-based therapies [292, 329]. Durable remission was observed in a DHL-BCL6 case when treated with lenalidomide following salvage chemotherapy [330]. Hence, more and larger clinical trials may be beneficial.

Bortezomib (PS-341) is the first generation of protease inhibitor with Ki of 0.6 nM, which shows good selectivity for tumor cells (Fig. 4) [331]. Bortezomib can prevent the degradation of IkB, keeping NF‑κB in its inactive form [332]. In the R/R DLBCL population, bortezomib alone did not bring great efficacy, but when combined with chemotherapy (DAEPOCH-R), the combination achieved better efficacy (response rate to 83% vs 13%; *P* < 0.001) and overall survival (10.8 vs 3.4 months; *P* = 0.003) in ABC vs GC DLBCL [333]. More recently, results of the randomized phase II PYRAMID study revealed no benefit to the use of bortezomib as an adjunct to R-CHOP in the frontline setting for non-GC DLBCL patients [334]. In a word, its clinical application is limited due to short drug effect time and severe dose-dependent neurological side effects [335].

Compared with the first generation of proteasome inhibitors, the second generation has oral biological activity, improved pharmacokinetics, and reduced neuropathy. Ixazomib is the first proteasome inhibitor that can be administered orally [336]. At present, preclinical experiments have proved that ixazomib has not only anti-tumor activity in DLBCL cell lines, but also inhibitory activity in DLBCL cell lines that have translocation of *MYC* and *BCL2*, and its IC_50_ value is 40-200 nm. A DHL xenograft mouse model showed anti-tumor activity at a clinically achievable drug concentration. At a dose of 7 mg/kg, the maximum tumor inhibition rate (TGI_max_) is 64%. Therefore, ixazomib is a potential drug for the treatment of DHL-BCL2, and its combination with CHK2 inhibitors provides a potential treatment option for drug-resistant DLBCL and DHL-BCL2 [337].

B-AP15 is a specific inhibitor of the deubiquitinating enzymes, and subsequently, inhibits the migration of DLBCL tumor cells and induces apoptosis. Preclinical experiments have shown that B-AP15 promotes apoptosis by inhibiting the activity of proteasome DUB (USP14 and UCHL5) in GCB and ABC-DLBCL cell lines [338], and inhibits WNT/β-catenin and TGFβ/Smad pathways to prevent DLBCL tumor cells migration. B-AP15 directly inhibits c-MYC protein, rather than inhibiting BCL2, but it can cause a decrease in BCL2 mRNA levels. Therefore, b-AP15 is not only a potential treatment for DLBCL but also DHL-BCL2 [298].

#### Targeting p53 signaling

Although 50% of tumors carry *TP53* mutations, the development of p53-targeted therapies is challenging [339]. Some *TP53* mutants do not encode a complete protein, and p53 itself, as a transcriptional factor, does not have an "active pocket" for small molecules to bind [340]. Current strategies targeting p53 mainly include targeting mutant p53 [341], synthetic lethality, and targeting MDM2-dependent p53 degradation [342]. However, these agents are only tested in leukemia, while studies in lymphoma remain unperformed [343–345].

XPO1 is a nuclear export receptor, which is involved in the nuclear-cytoplasmic transport of proteins bearing nuclear export signal (NES), and multiple RNA species [346]. XPO1 is usually overexpressed and/or mutated in the DLBCL cell lines, which causes poor nuclear retention of several proteins including several tumor suppressors like p53, BRCA1/2, and p27 [346, 347]. In addition, clinical research shows that high XPO1 expression has a significant adverse prognostic impact in DHL patients, especially in those with BCL2 overexpression [348]. Inhibition of XPO1 can down-regulate the expression of MYC in a variety of DHL cell lines [349].

Selinexor (KPT-330) is a first-in-class oral, biologically effective, and selective XOP1 inhibitor, which has been approved by the FDA for the treatment of heavily pre-treated R/R DLBCL and R/R multiple myeloma (Fig. 4) [299]. Selinexor can effectively down-regulate MYC protein expression and thus reprogram the gene expression of MYC downstream. Combined use of XPO1 and BCL2 inhibitors can synergistically induce the apoptosis of DHL tumor cells in vitro, and most importantly, block tumor progression and spread in vivo [349]. The effect of Selinexor has been confirmed in high-risk molecular DLBCL (both GCB and non-GCB subtypes), de novo, and transformed DLBCL and double-hit lymphomas [350].

#### Targeting cell cycle

CDKN2A is a tumor suppressor gene located on chromosome 9, encoding p16CDKN2A protein, which can inhibit the activity of CDK and regulate the G1 cell cycle [351]. Inactivation of CDKN2A may result in uncontrolled cell growth and proliferation. CDKN2A is frequently deleted, mutated, or hypermethylated in many tumors, including T-cell lymphoma, and is believed to be the second most commonly inactivated gene in cancer after p53 [351]. It is generally thought that CDKN2A gene loss-of-function mutations may lead to abnormal activation of CDK2/4/6, so CDK inhibitors may be a potential treatment for patients with CDKN2A mutations [352]. In addition, the same highly frequent alteration of the CCND3 gene was observed in MYC-BCL6 DHL patients, and the high expression of CCND3 can lead to downstream CDK4/6 activation, resulting in abnormal cell proliferation [353]. Therefore, CDK inhibitors may serve as potential therapeutic agents against MYC-BCL6 DHL.

Both palbociclib and abemaciclib are orally selective small-molecule CDK4/6 inhibitors that inhibit the progression of the cell cycle from G1 to S phase and block DNA synthesis [354]. A recent study found that both palbociclib and abemaciclib play a role in high-grade B-cell lymphoma with abnormal expression of CCND3 [355]. Pan-CDK inhibitor dinaciclib is proved to play synergistic induction of apoptosis in high-risk DLBCL when in combination with BCL2 inhibitor venetoclax [234]. In addition, CDK inhibitors such as flavopiridol and seliciclib were also found to be effective in BCL [356, 357], suggesting that targeted inhibition of CDK activity may indeed be a feasible treatment for DHL-BCL6. It is worth mentioning that a series of PROTACs studies targeting CDK has been progressively reported recently [358, 359], which may provide more treatment options for DHL.

#### Targeting CXCR4

As mentioned in the section of “3.1.6 Alterations relating cell migration”, the activation of the CXCR4 axis leads to cell dissemination to extranodal location. Two major strategies to target CXCR4 are direct inhibition and combination agents, aiming to induce cell death in CXCR4 + tumor cells.

Direct CXCR4 antagonists, including AMD3100(Plerixafor), AMD070, and WKI, demonstrated a pro-apoptotic effect in CXCR4 + DLBCL cells via modulation of JNK, ERK, NF-κB/ BCR, and BCL2 targets [176]. PF-06747143, a CXCR4 inhibiting IgG1 antibody, inhibits the signaling pathway and cell migration of lymphoma cells, reducing the bone marrow infiltration of Burkitt’s lymphoma in vivo [301]. In light of this, the safety and effect of CXCR4 targeted agents have been tested in human subjects in clinical trials. However, instead of cytotoxic agents, CXCR4 targeted drugs were mostly used as conditioning regimens. AMD3100, an extensively studied CXCR4 antagonist, was approved to mobilize hematopoietic stem cells for ASCT in NHL patients combined with G-CSF.

For conjugated agents, the liganded drug achieved higher tumor delivery and uptake than the free drug [360, 361]. T22-AUR is an auristatin E nanoconjugate that selectively targets CXCR4-overexpressing DLBCL cells. The cytotoxic effect of T22-AUR to CXCR4 + cells is significantly higher than free auristatin E since the CXCR4-dependent internalization of the conjugates facilitates the binding of auristatin E to tubulin, where auristatin E induces cell death. The use of T22-AUR in DLBCL xenograft reduced the dissemination of tumor cells into bone marrow and the central nervous system [302].

One clinical study was initiated recently to investigate the safety of CXCR4 modified CD19 CAR-T cells in refractory B-cell NHL (NCT04684472). Another preclinical study recruited six patients with heavily pretreated DLBCL. They were administered with CXCR4-directed radioligand therapy in combination with conditioning chemotherapy and allogeneic stem cell transplantation. The regimen was well tolerated, resulting in CR of all-CXCR4 + lymphoma but the appearance of CXCR- lesions, supporting the use of CXCR4-directed radioligand therapy as a conditioning regimen [303].

In all, CXCR4 antagonists can reduce tumor burden and the dissemination of lymphoma to organs. However, the use of these regimens is limited to CXCR4 + lymphomas like CXCR4 + disseminated refractory or relapsed DLBCL patients. For a broader spectrum of patients, the combination of CXCR4 antagonist and other therapy (chemotherapy, targeted therapy, or radiotherapy) is needed.

#### Targeting immune escape

Recurrent mutations associated with immune escape genes in the DHL-BCL6 may suggest that this subtype is a better candidate for immunotherapy than DHL-BCL2. A number of ongoing trials are investigating the monotherapy and combination therapy of anti-PD-1 antibodies in DLBCL. The results of several clinical trials have been published.

In a phase Ib dose-escalation study of nivolumab, the ORR was 36% in R/R DLBCL with most adverse events (80%) under grade 3 [304]. However, in another phase II study, the ORR was lower, 10% and 3% in relapsed patients after or were ineligible for autologous hematopoietic cell transplantation, respectively. It is worth noting that the membranous PD-L1 level of DLBCLs in this study is only 9%, which may contribute to the differences in the two studies [305]. A study of 105 DLBCL patients showed that when the *PD-L1* gene is altered, increased PD-L1 protein downregulated HLA expression, activated NF-κB, and higher response to PD-1 blockade (anti-PD-1 antibody pembrolizumab) were observed (NCT01953692) [306]. Another study observed a similar result when treatment naïve DLBCL patients were treated with pembrolizumab in combination with R-CHOP. The toxicity was comparable to R-CHOP alone. The ORR was 90% and the CR rate was 77%. Similar to the study above, higher PD-L1 expression was associated with non-GCB subtype and improved PFS and survival [307]. Based on the study above, together with results in primary CNS lymphoma and primary testicular lymphoma [362], B cell lymphomas with PD-L1 amplification, gain or aberration are likely to have a promising response to PD-1 blockade therapy.

### Combined regimens for DHL

Although the targeted agents showed good efficacy or potential for the treatment of DHL with monotherapy, combined regimens had been evaluated on DHL patients, or specific clinical trials on DHL patients are ongoing, such as NCT04479267. We summarized therapeutic targets that had been under clinical evaluation with combined regimens, including the status of clinical trials, clinical response rate, adverse events and so on in Table 6. All these clinical trials covered DHL in the inclusion criteria or reported DHL cases in the relevant publications.

**Table 6:** Therapeutic targets that had been under clinical evaluation for DHL with combined regimens

Target	Agent	Therapeutics	Stage	Status	N^a^	Clinical response	Adverse events	References
BCL2	Venetoclax	+ DA-EPOCH-R	Phase II/III (high-grade B-cell lymphomas, including DHL)	Suspended (2019–2028)	–	No result posted	No result posted	NCT03984448
			Phase I (aggressive B-Cell Lymphomas, including DHL)	Active, not recruiting (2017–2021)	31	ORR: 96·7% (95% CI 82·8–99·9); complete response: 28 (93·3% [77·9–99·2]) of 30; partial response: 1 (3·3% [0·1–17·2])	Grade 3–4 adverse events: cytopenias (28 [93%] of 30 patients); febrile neutropenia occurred in 19 (63%) patients. Grade 3–4 non-haematological adverse events included hypophosphataemia (*n* = 10), hypokalaemia (*n* = 7), and hyperglycaemia (*n* = 5) Serious adverse events: infection (*n* = 7) and gastrointestinal toxicities including abdominal pain (*n* = 3), colonic perforation (*n* = 1), and small intestinal obstruction (*n* = 1)	NCT03036904 [363]
Proteasome	Bortezomib	+ Cyclophosphamide, Dexamethasone, vincristine sulfate liposome	Phase II (r/r ALL, LL, Burkitt Lymphoma, DHL)	Recruiting (2017–2026)	–	No result posted	No result posted	NCT03136146
		+ R-CHOP or RB-CHOP	Phase III (DLBCL and other B cell lymphoma, including DHL)	Completed (2011–2015)	1132 (18 DHL)	Clinical response (DHL): + R-CHOP: 30-months PFS, 38.9%; + RB-CHOP: 30-months PFS, 58.8%; 30-months OS, DHL:DEL:DLBCL, 38·9% vs 61·5% vs75·8%	Most common AEs: Neutropenia, thrombocytopenia, and neuropathy	NCT01324596 [364]
CD52	Alemtuzumab	+ Cyclophosphamide	Phase I (Aggressive lymphoma, including DHL)	Terminated (slow accrual)	3	No result posted	No result posted	NCT03132584
CD79B	Polatuzumab Vedotin	+ R-CHOP or R-CHP	Phase II (DHL, THL)	Recruiting (2020–2022)	–	No result posted	No result posted	NCT04479267
HDAC	Tucidinostat	+ BEAC	Phase II (aggressive lymphoma, including DHL)	Active, not recruiting (2018–2021)	69	No result posted	No result posted	NCT03629873
	Fimepinostat	Monotherapy, or + rituximab	Phase I (r/r DLBCL, HGBL, including DHL)	Completed (2012–2020)	106^b^	Monotherapy: OR 71%, mDOR 13.6 months, mPFS 21.8 months; + rituximab: OR 50%, ORR 64%,	Most common: diarrhea [21 (57%)], thrombocytopenia [20 (54%)], fatigue [15 (41%)], nausea [14 (38%)], constipation [9 (24%)], vomiting [9 (24%)], and neutropenia [8 (22%)]; Grade ≥ 3 adverse events:16 (43%) patients, thrombocytopenia [12 (32%)], neutropenia [6 (16%)], anemia [2 (5%)], diarrhea [2 (5%)], and fatigue [2 (5%)]	NCT01742988 [203, 365]
	Chidamide	+ ChiCGB and auto-SCT	Phase II (r/r DLBCL)	Completed (2017–2021)	93 (2 DHL)	Clinical response: DHL DLBCL: 8%; 4-year PFS 90.0%, 4-year OS 96.8%	Most frequent AEs: Mucositis (43.8%), dermatitis (33.3%), transaminase elevation(43.8%)	NCT03151876 [366]
CD3/CD20	Epcoritamab	+ BR or R-GemOx	Phase III (r/r DLBCL, DHL/THL, FL grade 3B)	Recruiting (2021–2024)	–	No result posted	No result posted	NCT04628494
PD-1	Nivolumab	+ DA-EPOCH-R	Phase II (DHL/THL-HGBL)	Recruiting (2018–2026)	–	No result posted	No result posted	NCT03620578
	Pembrolizumab	+ DPX-Survivac, Cyclophosphamide	Phase II (r/r DLBCL, HGBL, including DHL)	Active, not recruiting (2018–2022)	25	No result posted	No result posted	NCT03349450
CRBN	Lenalidomide	+ DA-EPOCH-R	Phase II (lymphoma including DLBCL)	Active, not recruiting (2014–2022)	15 (5 DHL)	Clinical response: 13 CRs (87%), 1 PR (7%), and 1 case of PD (7%), 2-year OS rate87% and PFS rate 87%	Most common Aes: thromboembolism (4 patients; 27%) and hypokalemia (2 patients; 13%) SAEs:	NCT02213913 [292]
DNMT1/2	Azacitidine	+ R-miniCHOP	Phase II/III (DLBCL, Grade 3B FL, DHL/THL-HGBL)	Recruiting (2021–2025)	–	No result posted	No result posted	NCT04799275 [367]
		+ R-CHOP	Phase I (DLBCL, FL, or Transformed lymphoma)	Completed (2015–2020)	59 (2 DHL, 1THL)	Dosage: 100-300 mg daily Clinical response: ORR, 94.9%; CR, 88.1%; estimated 1-year PFS, 84.1%; estimated 2-PFS, 78.6%	MTD was not reached; 2 DLTs Most common grade 3/4 toxicities: Neutropenia (62.7%) and febrile neutropenia (25.4%)	NCT02343536 [368]
XPO1	Selinexor	+ R-CHOP	Phase Ib/II (r/r DLBCL, DHL)	Recruiting (2017–2023)	12^b^	Dosage: 60/80 mg daily Clinical response: CR, 80%; PR, 10%	Most common Aes: nausea (100%), fatigue (67%), skin/nail changes (58%), vomiting (42%), dizziness (42%), sinus congestion (42%), and constipation (42%)	NCT03147885 [369]
		+ Choline Salicylate	Phase Ib (DLBCL, MCL, DHL)	Recruiting (2021–2024)	–	No result posted	No result posted	NCT04640779
		+ RICE	Phase I (aggressive B-Cell Lymphoma, including DHL)	Active, not recruiting (2015–2021)	22	No result posted	No result posted	NCT02471911
mTOR	Everolimus	Maintenance therapy with Rituximad	Phase II (Lymphomas)	Completed (2018–2021)	56 (1 DHL)	30 month OS: 93%; overall median EFS: 36.4 months; 30 months EFS: 58%	Most frequent AEs: grade 3/4 neutropenia (61.2%), hyperglycemia (18.4%), hypertriglyceridemia (18.4%), thrombocytopenia (16.3%), and anemia (8.2%)	NCT01665768 [370]

^a^Actual cases or reported cases

^b^No DHL case was reported in the publication

## Conclusions and future perspectives

Due to lack of effective treatment and late diagnosis, DHL patients are subjected to intensive chemotherapy and poor outcome. To avoid the late diagnosis, the study result supports the performance of the routine FISH test in all DLBCL patients, which considerably improves the outcome of DHL (ST vs RT: 2-year RFS, 38% v 70% and 2-year overall survival, 38% v 74%). At the same time, routine testing identified a cohort of low-risk DHL patients and the lack of benefit from intensive immunochemotherapy [28]. Independent research further stratifies DHL into high-risk and low-risk (DHIT + SIG) subgroups, suggesting tremendous heterogenicity lies in the DHL cohort [26]. However, despite the satisfiable efficacy of standard R-CHOP in low-risk DHL, the poor outcome of high-risk DHL remains a challenge, while dose-adjusted intensive regimens become appropriate induction options for most patients with double-hit lymphoma in clinical practice.

To facilitate risk stratification and treatment selection, approaches have been applied to classify DLBCL into different subgroups. Based on cell-of-origin, DLBCLs can be categorized into GCB and non-GCB types, which further include ABC DLBCL and unclassifiable DLBCL. These subgroups differ in biological features, clinical outcomes, and treatment response. For instance, GCB-DLBCL tends to present genetic alterations in the PI3K pathway, which are related to “tonic” BCR signaling, while ABC-DLBCL was observed to harbor mutations characterized as chronic active BCR signaling [143, 371]. Hence, this fundamental divergence contributes to the different responses of DLBCL subtypes to NF-κB pathway inhibition [327]. Similarly, high sensitivity to EZH2 inhibitors was also observed in DLBCLs with hyper-H3K27me3 status, which is closely related to the GCB cohort [372].

Considering heterogenicity in the DLBCL group, we aim to link distinct genetic events to different DLBCL subgroups, i.e. DHL-BCL2 and DHL-BCL6. After extensive literature, we summarized unique genetic events encompassed in the DHL subtypes. We further collected potential agents targeting these abnormalities, hopefully providing insights into the treatment of DHL. However, this review has certain limitations. Owing to the dominant proportion in DHL and close coincidence with GCB DLBCL, mutations and treatment strategies concerning the DHL-BCL2 cohort are far more elaborate than the DHL-BCL6, which was the minority of DHL with a mixed composition of GCB, ABC, and unclassifiable DLBCLs. More studies and investigations should be done to this subtype, as the incidence of DHL-BCL6 in east Asia is considerable (comparison of two centers: South China: DHL-BCL2 (37%), DHL-BCL6 (63%) [141] vs North American: DHL-BCL2 (87%), DHL-BCL6 (23%) [32].

Although alteration of some pathways was repeated spotted in DHL, agents targeting corresponding proteins or pathways were rarely tested in DLBCLs or yielded unsatisfactory results. As more studies highlight the unique signaling in DHL, novel agents or therapeutic strategies, like CAR-T and CAR-NK, can be tested to accelerate treatment development for these patients.

## Data Availability

The material supporting the conclusion of this review has been included within the article.

## Abbreviations

ARID1A: AT-rich interaction domain 1A
AICDA: Activation-induced cytidine deaminase
ASCT: Autologous stem cell transplant
BAK: BCL2 homologous antagonist killer
BAX: BCL2-associated X protein
BCL: B-cell lymphoma
BCR: Breakpoint cluster region protein
BET: Bromodomain and extraterminal protein
BFL: BCL2-related protein A1
BIRC5: Baculoviral IAP repeat containing 5
BRD4: Bromodomain containing 4 protein
BTK: Bruton's tyrosine kinase
CAR-T: Chimeric antigen receptor T-cell
CDK4/6: Cyclin dependent kinase 4 and 6
CHK2: Checkpoint kinase 2
CR: Complete response
CRBN: Cereblon
CREBBP: CAMP response element-binding protein
CXCR4: C-X-C motif chemokine receptor 4
DEL: Double-expressor lymphoma
DHL: Double-hit lymphoma
DLBCL: Diffuse large B-cell lymphoma
DSS: Disease-specific survival
EBF1: Early B cell factor 1
EFS: Event-free survival
ERK: Extracellular signal-regulated kinase
EZH2: Enhancer of zeste homolog 2
FAS: Fas cell surface death receptor
FISH: Fluorescence in situ hybridization
FOXO1: Forkhead box O1
FLT3: Fms related receptor tyrosine kinase 3
GCB: Germinal center B-cell like
GEP: Gene expression profile
GNA13: G-Protein subunit alpha 13
HES1: Hes family BHLH transcription factor 1
HDAC: Histone deacetylase
HLA-DMB: Major histocompatibility complex, class II, DM beta
HVCN1: Hydrogen voltage gated channel 1
IAP2: Inhibition of apoptosis protein 2
IFRT: Involved-field radiation therapy
IRF: Interferon regulatory factor
IKK: IκB kinase
IMiD: Immunomodulatory imide drug
JAK: Janus kinase
KMT2D: Lysine methyltransferase 2D
MCL1: Induced myeloid leukemia cell differentiation protein
MEF2B: Myocyte enhancer factor 2B
mTOR: Mammalian target of rapamycin
MYC: MYC proto-oncogene
NF-κB: Nuclear factor kappa-light-chain-enhancer of activated B cells
NHL: Non-Hodgkin's lymphoma
NMT: N-myristoyltransferase
OCT2: Octamer-binding protein 2
ORR: Overall response rate
OS: Overall survival
PFS: Progression-free survival
PI3K: Phosphoinositide 3-kinase
PIK3R1: Phosphoinositide-3-kinase regulatory subunit 1
PKC: Protein kinase C
POU2F2: POU class 2 homeobox 2
PTEN: Phosphatase and tensin homolog
PLK1: Polo like kinase 1
REL: Proto-oncogene c-Rel
RFS: Relapse-free survival
R-CHOP: Rituximab, cyclophosphamide, doxorubicin, vincristine, prednisone
R-DA-EPOCH: Rituximab, etoposide, prednisone, vincristine, cyclophosphamide, doxorubicin
R-Hyper-CVAD: Rituximab, hyperfractionated cyclophosphamide, vincristine, doxorubicin, dexamethasone, alternating with cytarabine plus methotrexate
R-CODOX-M/IVAC: Rituximab, cyclophosphamide, vincristine, doxorubicin, methotrexate/ ifosfamide, etoposide, cytarabine
STAT: Signal transducer and activator of transcription
SMAC: Second mitochondria-derived activator of caspase
SRSF2: Serine/arginine-rich splicing factor 2
SIPR2: Sphingosine-1-phosphate receptor 2
SYK: Spleen associated tyrosine kinase
TEFB: Transcription elongation factor b
TGFβ: Transforming growth factor beta
THL: Triple-hit lymphoma
TNFRSF14: TNF receptor superfamily member 14
TNFR1: Tumor necrosis factor receptor 1
TYK2: Tyrosine kinase 2
XPO1: Exportin 1
